# A U3 snoRNA is required for the regulation of chromatin dynamics and antiviral response in *Drosophila melanogaster*

**DOI:** 10.1093/nar/gkaf715

**Published:** 2025-07-30

**Authors:** Shruti Jain, Jordi Planells, Isabel Regadas, Donal Barrett, Anne von Euler, Indranil Sinha, Bo Gustav Lindberg, Jenny C Hesson, Patrycja Swacha, Nelson O Gekara, Vicent Pelechano, Ylva Engström, Mattias Mannervik, Neus Visa

**Affiliations:** Department of Molecular Biosciences, Wenner-Gren Institute, Stockholm University, Stockholm SE-106 91, Sweden; Department of Molecular Biosciences, Wenner-Gren Institute, Stockholm University, Stockholm SE-106 91, Sweden; Department of Molecular Biosciences, Wenner-Gren Institute, Stockholm University, Stockholm SE-106 91, Sweden; SciLifeLab, Department of Microbiology, Tumor and Cell Biology, Karolinska Institutet, Solna SE-171 65, Sweden; Department of Molecular Biosciences, Wenner-Gren Institute, Stockholm University, Stockholm SE-106 91, Sweden; Department of Molecular Biosciences, Wenner-Gren Institute, Stockholm University, Stockholm SE-106 91, Sweden; Department of Molecular Biosciences, Wenner-Gren Institute, Stockholm University, Stockholm SE-106 91, Sweden; Department of Medical Biochemistry and Microbiology, Zoonosis Science Center, Uppsala University, Uppsala SE-751 23, Sweden; Department of Molecular Biosciences, Wenner-Gren Institute, Stockholm University, Stockholm SE-106 91, Sweden; Department of Molecular Biosciences, Wenner-Gren Institute, Stockholm University, Stockholm SE-106 91, Sweden; SciLifeLab, Department of Microbiology, Tumor and Cell Biology, Karolinska Institutet, Solna SE-171 65, Sweden; Department of Molecular Biosciences, Wenner-Gren Institute, Stockholm University, Stockholm SE-106 91, Sweden; Department of Molecular Biosciences, Wenner-Gren Institute, Stockholm University, Stockholm SE-106 91, Sweden; Department of Molecular Biosciences, Wenner-Gren Institute, Stockholm University, Stockholm SE-106 91, Sweden

## Abstract

Small nucleolar RNAs (snoRNAs) are prevailing components of the chromatin-associated transcriptome. Here we show that specific snoRNAs are required for the activation of immune response genes and for survival during viral infections in *Drosophila melanogaster*. We have studied *snoRNA:U3:9B*, a chromatin-associated snoRNA that binds to a large number of protein coding genes, including immune response genes. We have used CRISPR/Cas9 to delete *snoRNA:U3:9B* and study its function *in vivo*. *SnoRNA:U3:9B*-deficient larvae are viable but failed to develop into pupae when challenged by expression of a Sindbis virus replicon. *SnoRNA:U3:9B* is localized to immune genes *in vivo* and the chromatin decompaction and gene activation typically observed at immune genes following infection are abolished in *snoRNA:U3:9B*-deficient larvae, which suggests that this snoRNA acts locally to regulate chromatin accessibility. Mechanistically, *snoRNA:U3:9B* is required for the recruitment of the chromatin remodeler Brahma to a set of target immune genes. In summary, these results uncover an antiviral defense mechanism that relies on a snoRNA for the recruitment of a chromatin remodeling factor to immune genes to facilitate immune gene activation.

## Graphical Abstract

**Graphical Abstract:**
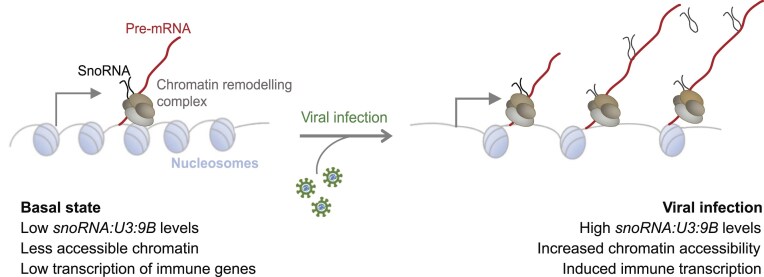

## Introduction

Noncoding RNAs (ncRNAs) play important regulatory functions. Some ncRNAs are exported to the cytoplasm, but others can associate with the chromatin and regulate genome architecture and gene expression [1]. The study of the chromatin–RNA interactome has been boosted by the development of technologies for global profiling of chromatin-associated RNAs (caRNAs) [2–6]. These technologies have provided comprehensive descriptions of the chromatin-associated transcriptome and have revealed that small nucleolar RNAs (snoRNAs) constitute a relatively large caRNA component in both insect and mammalian cells [7, 8]. SnoRNAs are small ncRNAs with a length of 60–300 nt that are conserved among eukaryotes [9]. They associate with a set of proteins to form snoRNA–protein complexes (snoRNPs), and their canonical function is to guide site-specific modifications in a variety of target RNAs through interactions that rely on sequence complementarity [10–12]. The RNA targets of many snoRNAs have been either identified experimentally by methods developed for detection of RNA–RNA interactions [13–16] or predicted with the help of *in silico* approaches [17–19]. The fly and human genomes encode ∼250 and 750 snoRNA genes, respectively [20, 21].

SnoRNAs were initially discovered for their roles in the processing and modification of rRNAs, but they are also responsible for post-transcriptional modifications of other RNA species, including small nuclear RNAs (snRNAs), transfer RNAs, and messenger RNAs (mRNAs) [22–24]. From a structural perspective, snoRNAs can be classified into two main groups that are characterized by distinctive structural elements or boxes, specific sets of associated proteins, and the type of chemical modification they direct. The box C/D snoRNAs associate with the methyltransferase Fibrillarin and guide 2′-O-ribose methylation, while the box H/ACA snoRNAs interact with Dyskerin and direct pseudouridylation of their target RNAs [11]. Moreover, some snoRNAs such as the U3 snoRNAs act by promoting site-specific pre-ribosomal RNA (rRNA) cleavage rather than guiding nucleotide modifications [25].

In addition to their housekeeping functions in processing ribosomal RNA precursors, snoRNAs have been implicated in a variety of other cellular processes. These include mitotic progression, telomere maintenance, regulation of pre-mRNA splicing, and 3′ end processing [26–29]. Moreover, some snoRNAs are processed into miRNA-like molecules that can act like miRNAs [30] and others have regulatory functions. For example, snoRNPs activate PARP-1 in breast cancer cells, which enhances ribosome biogenesis and cell proliferation [31], and a small Cajal body-associated snoRNA regulates DNA repair by binding to the catalytic subunit of the DNA-dependent protein kinase DNA-PK [32]. SnoRNAs have also been involved in the regulation of chromatin compaction. Following a series of elegant *in vitro* experiments, Schubert *et al.* [33] showed that snoRNAs together with the histone binding protein Df31 can bind chromatin and relax higher-order chromatin structures in both human and insect cells. However, the extent of such regulation *in vivo* and its physiological significance remain largely unexplored.

Given their crucial roles in RNA biology, it is not surprising that snoRNAs are key players in human diseases, as demonstrated by research conducted over the past decade. For example, loss of the human *SNORD116* gene has been directly linked to the Prader–Willi syndrome [34, 35], mutations in the *U8 snoRNA* cause Labrune syndrome, a neurological disorder that affects cerebral small blood vessels [36], and several other snoRNAs have been proposed to act as proto-oncogenes or tumor suppressors, which has sparked interest in snoRNAs as potential targets for diagnostics or therapeutic interventions [37–41]. Moreover, snoRNAs are often deregulated upon viral infection and many of them have been found to be involved in viral replication or virus-host interactions [42].

*Drosophila melanogaster* is a powerful model organism to study innate immune responses [43, 44]. The *Drosophila* antiviral defense includes cellular reactions and humoral responses that involve the production of antimicrobial peptides (AMPs) and other immune effectors. RNA interference is the major antiviral defense mechanism in insects and operates against a large diversity of viruses [45, 46]. In the RNA interference pathway, the ribonuclease Dicer-2 recognizes double-stranded RNAs of viral origin and cleaves them into small interfering RNAs that are loaded onto RISC complexes and enable RISC to degrade complementary viral sequences. Dicer-2 also induces the expression of Vago, a small antiviral peptide that activates the JAK-STAT pathway [47]. JAK-STAT, together with Toll, Imd, MAPK/JNK, and Hippo, are evolutionarily conserved signaling pathways that contribute to insect innate immunity in different ways [48, 49]. The Toll and Imd pathways, which are essential for the immune response to fungi and bacteria via the production of AMPs [50], also play a role in antiviral defence through mechanisms that remain poorly understood [51, 52]. The MAPK/JNK and Hippo pathways are involved in a variety of biological processes and contribute to the innate immune responses by modulating the expression of key Toll and Imd factors [53, 54]. Recent research has demonstrated that the coordinated action of various immune pathways is a critical feature of the antiviral response in *Drosophila* [55].

We have profiled caRNAs in Schneider 2 (S2) cells of *D. melanogaster* [56] and identified several snoRNAs, including *snoRNA:U3:9B*, that are enriched in the chromatin. Here we combine molecular biology and loss-of-function approaches to study the role of *snoRNA:U3:9B*. This snoRNA shares a high degree of sequence homology with two other *U3 snoRNAs* that play an essential role in pre-rRNA processing [57]. However, *snoRNA:U3:9B* contains a unique RNA sequence that is not present in the other *U3 snoRNAs* (Supplementary Fig. S1). *SnoRNA:U3:9B* is expressed at relatively low levels under normal growth conditions and genetic knock-out flies that lack the *snoRNA:U3:9B* gene are viable. The expression of *snoRNA:U3:9B* is significantly upregulated following infection with Sindbis virus and that deletion of the *snoRNA:U3:9B* gene is lethal in combination with Sindbis virus infection. While other snoRNAs are necessary for optimal viral replication, our results reveal that *snoRNA:U3:9B* is required for the activation of antiviral response genes through a mechanism that involves changes in chromatin accessibility.

## Materials and methods

### Culture of S2 cells

*Drosophila melanogaster* S2 cells were cultured at 28°C in Schneider’s medium (Gibco) supplemented with 10% fetal bovine serum (FBS; Gibco), 50 μg/ml streptomycin, and 50 U/ml penicillin (Gibco).

### Total RNA and caRNA extractions

Total RNA was extracted using Trizol (Ambion) and ethanol-precipitated following standard procedures. Isolated RNA was quantified using NanoDrop One (Thermo Scientific). For caRNA extraction, S2 cells were crosslinked with formaldehyde for 10 min at room temperature (RT) and the cross-linking was stopped by addition of 1 M glycine for 10 additional min. The cells were then collected and resuspended in Buffer 1 [50 mM Hepes, pH 7.6, 140 mM NaCl, 1 mM ethylenediaminetetraacetic acid (EDTA), 10% glycerol, 0.5% NP-40, 0.25% Triton X-100, and cOmplete protease inhibitors] and incubated for 10 min at 4°C, followed by centrifugation at 4°C and 500 × *g*. The pellet was resuspended in cold Buffer 2 (200 mM NaCl, 1 mM EDTA, 0.5 mM ethyleneglycol tetraacetic acid (EGTA), 10 mM Tris, pH 8, and cOmplete protease inhibitors) and centrifuged again. Finally, chromatin was resuspended in Buffer 3 (1 mM EDTA, 0.5 mM EGTA, and 10 mM Tris, pH 8, and cOmplete protease inhibitors). Chromatin was fragmented using a Bioruptor sonicator (Diagenode) with 40 high intensity sonication 30 s on/off pulses. Protein concentration was measured using a NanoDrop One. The chromatin fraction was treated with DNAse I (Thermo Scientific) and Proteinase K (Thermo Scientific). RNA was isolated with Trizol (Ambion). RNA concentration was measured with NanoDrop.

### Reverse transcription quantitative polymerase chain reaction (RT-qPCR)

RNA was extracted with TRIzol reagent (Ambion, Thermo Fisher), treated with 1 unit DNase I (Thermo Fisher) for 60 min and reverse-transcribed using random primers (Thermo Fisher Scientific) and SuperScript III (Invitrogen). The resulting complementary DNAs were used for quantitative polymerase chain reaction (qPCR) using KAPA SYBR Fast qPCR Master Mix (Kapa Biosystems) in a BioMolecular Systems MIC instrument. Primer design was according to MIQE guidelines. All primer pairs fulfilled quality criteria regarding amplification efficiency and melting curves. The primer sequences are listed in Supplementary Table S1. The polymerase chain reaction (PCR) primers used for analysis of *snoRNA:U3:9B* are also shown in Supplementary Fig. S1. The results presented are compiled data from multiple independent biological replicates, each analysed in duplicate. For each experiment, the number of independent replicates is provided in the figure legend. For analysis of snoRNA abundance in knock-out strains where the sequences analyzed are undetectable, the maximum number of 40 cycles has been used for delta Ct calculations.

### RNA fluorescence *in situ* hybridization and immunofluorescence

Larval salivary glands were dissected and fixed with 3.6% formaldehyde in phosphate-buffered saline (PBS) containing 1% Triton X-100 for 40 s followed by 2 min in 3.6% formaldehyde/50% acetic acid solution. The glands were squashed in lactoacetic acid (lactic acid:water:acetic acid; 1:2:3) and the coverslips flipped-off after freezing the preparations in liquid nitrogen. Chromosome squashes we prehybridized with 10 μl hybridization buffer (HB) containing 2× saline sodium citrate (SSC) buffer, 50% formamide and 5% dextran sulfate for 30 min at 42°C. Probe mix was freshly prepared and contained 1.2 μl probe at 1.2 μg/ml, 0.4 μl salmon sperm DNA (10 mg/ml), and 8.4 μl HB. Hybridization was for 1 h at 42°C. The slides were washed sequentially with the following solutions for 3 min each: 50% formamide at 42°C, 5× SSC at 42°C, 2× SSC at 42°C, 0.1× SSC at room temperature, and 1× PBS/10% Tween 20 (PBT) at room temperature. The slides were then blocked with 3% bovine serum albumin (BSA) in PBT for 45 min and incubated with primary antibody (anti-DIG-FITC; Roche, 11209941910). After washing in PBT twice for 3 min each, slides were incubated with secondary antibody (anti-FITC- Alexa488, Molecular Probes, A11090) for 40 min at 37°C, washed again with PBT and mounted using 10 μl Vectashield with DAPI (4′,6-diamidino-2-phenylindole, Vector Laboratories, H-1200-10). The preparations were counterstained with antibodies against either H3K9ac (Abcam, ab10812), Df31 (kind gift from Jordan Rowley, University of Nebraska Medical Center), RNApolII (Abcam, ab5408) or Fibrillarin (Abcam, ab5821). The slides were examined in an Axioplan fluorescence microscope (Carl Zeiss). The probe sequences are listed in Supplementary Table S1 and shown in Supplementary Fig. S1.

### Bioinformatics analyses

RNA-seq data was obtained from Planells *et al.* [56] [GEO accession GSE222262]. Normalized reads (transcripts per million, TPM) were computed individually for each replicate and averaged (N = 3). Transcript biotype information was obtained from the ensembl BDGP6.28.100 reference gene annotation. ChAR-seq [3] [GEO accession GSE97131] RNA–DNA contacts mapped to *D. melanogaster* dm3 assembly were converted to dm6 using rtracklayer::liftOver function in Bioconductor and the UCSC chain file (dm3Todm6). Exon-intron assignment of ChAR-seq RNA–DNA contacts was performed using the R package annotatr [58].

Gene ontology analyses were carried out using ShinyGO 0.81 [59]. Motif analysis was performed using the MEME suite. The 500 bp sequence upstream of transcription start site (TSS) of chromatin-associated snoRNAs (ca-snoRNAs) was obtained using ensembl biomart and uploaded to the XSTREME tool [60] under the MEME suite. SnoGloBe [19] was used to predict RNA–RNA interactions of *snoRNA:U3:9B*, *snoRNA:185*, and *snoRNA:28S-A2468* against the entire transcriptome of the Ensembl BDGP6.46.110 assembly release (excluding chromosome scaffolds), using the following settings: -n 10 -t 0.95 -m -w 3 –seq. The STREME tool [61] under the MEME suite was used to identify novel motifs in the *snoRNA:U3:9B* target sequences predicted by snoGlobe.

### *Drosophila* stock maintenance

All fly stocks were reared on instant potato mash–agar food in mixed female/male populations at 25°C, 60% relative humidity, and a 12-h light/12-h dark cycle.

### Microbial infections

Bacterial overnight cultures of *Entercoccus faecalis* or *Micrococcus luteus* were washed once and resuspened in PBS at OD 1. A field isolated strain of Sindbis virus (genus Alphavirus, family Togaviridae), genotype I (09M-99[1]-1) was grown in Vero cells [62]. A vesicular stomatitis virus (VSV) strain, VSV-GFP, was grown in Vero cells as previously reported [63]. Late second instar larvae were injected with either 50 nl of bacterial suspension, 50 nl of 4.0e + 9 plaque forming units/ml (PFU/ml) VSV-GFP or 50 nl of 3.4e + 9 PFU/ml Sindbis virus through the cuticle towards the posterior end. Larvae injected with the same volume of either PBS or Dulbecco’s modified Eagle’s medium (DMEM; Gibco) supplemented with 10% FBS (Gibco) served as mock-infected controls. *w^1118^* larvae were used as background strain. Following injection, animals were maintained at 29°C. Five brains per replicate were harvested 24 h after injection for RNA extraction and RT-qPCR analysis.

### Sindbis infection model in flies

Transgenic flies expressing SINrep:GFP or SINΔrep:GFP were obtained from Richard Hardy, Indiana University, and crossed with *Act > GAL4* (BL#3954) stock to drive the expression of the *SIN* replicons.

### CRISPR/Cas9 alleles and generation snoRNA knock-outs

The gRNAs targeting *snoRNA:U3:9B* and *snoRNA:Me28S-A2486* were designed using the targetFinder tool (http://tools.flycrispr.molbio.wisc.edu/targetFinder/). Primers containing homology to the target genes (listed in Supplementary Table S1) were annealed and cloned into the pCFD4 plasmid [64] at the *BbsI* site using Gibson Assembly (NEB). The resulting plasmids were sequence verified, purified with the NucleoBond Xtra Midi kit (Macherey-Nagel, Cat. 740410.50) and inserted into the attP40 landing site (FlyORF Injection Service). Transgenic flies were identified by their *vermilion*+ eye phenotype. The knock-outs were generated by crossing with *vasa-cas9* fly stock and genetic screening was performed according to standard protocol (https://flycrispr.org/wp-content/uploads/2019/07/ssODN-molecular-screening-work-flow.pdf). The deletions were confirmed by genomic DNA PCR and Sanger sequencing.

### RNA sequencing and data analysis

RNA was extracted as described above, in triplicates. The siTOOLs riboPOOL kit was used for rRNA depletion. Paired-end strand-specific libraries were prepared using NEBNext Ultra II directional RNA library Prep Kit for Illumina (NEB) according to the manufacturer’s instructions. Total RNA-seq was performed by the core facility for Bioinformatics and Expression Analysis (Karolinska Institute, Huddinge, Sweden) on a NextSeq 2000 P2 (100 cycles, 2 × 58 bp) sequencer aiming for an average depth of 15 million reads per sample. RNA-seq data was processed using nf-core/rnaseq v3.12.0 [65]. The pipeline was executed with Nextflow v23.10.1 [66]. Briefly, raw RNA-seq reads were aligned to the *D. melanogaster* reference genome (dm6). The resulting BAM files were then imported into R (version 4.3.2) for further processing and differential expression analysis. Batch effects were corrected using Surrogate Variable Analysis. Differential gene expression was performed using DESeq2 [67] with models based on a negative binomial distribution, producing a final set of differentially expressed gene lists for downstream analyses (false discovery rate, FDR < 0.05).

### Assay for transposase-accessible chromatin-qPCR in S2 cells and third instar larval brains

Tagmentation reaction in S2 cells was performed according to assay for transposase-accessible chromatin (ATAC)-seq method [68] using Nextera DNA library kit (FC-121–1030). Tagmentation reaction from five brains dissected from third instar larvae was performed according to Dhall *et al.* [69]. The purified tagmented DNA was analyzed by ATAC-qPCR using the primers listed in Supplementary Table S1.

### ATAC sequencing and data analysis

The samples (in triplicates) were prepared as described above for ATAC-qPCR. Libraries were sequenced by the core facility for Bioinformatics and Expression Analysis (Karolinska Institute, Huddinge, Sweden) on NextSeq 2000 P3 (100 cycles) sequencer obtaining paired-end reads (2 × 61bp). The average depth was over 60 million reads per sample. ATAC-seq data was processed using the nf-core/atacseq v2.0 pipeline (https://nf-co.re/atacseq/2.0), executed with Nextflow v23.04.2. Briefly, raw FASTQ files were quality-checked with FastQC, adapters were trimmed with TrimGalore, and reads were aligned to the *D. melanogaster* reference genome (dm6) using BWA. Blacklisted regions [70] were excluded based on the dm6-blacklist.v2.bed. Differential accessibility was assessed using a window-based approach in the csaw package [71] in R (version 4.3.2). Three normalization methods—TMM, Lowess, and Full Quantile (FQ)—were tested, with FQ normalization yielding the most consistent results. Differential accessibility gene lists were generated for further analysis (FDR < 0.05).

### Sindbis infection model in S2 cells

The pSINrep:GFP, kindly donated by Richard Hardy, was digested with *RsrII* and *KpnI* to create the pSINΔ:GFP plasmid as described in Avadhanula *et al.* [72]. The plasmids were stably transfected into S2 cells using a calcium phosphate transfection kit (Invitrogen, cat. no. 44-0052) and hygromycin selection. Expression of the *SIN* replicon in stable cell lines was induced by transfection of pAc-GAL4 (Addgene, #24344) following standard procedures for transient transfection. The cells were harvested and analyzed 48 h after transfection.

### SnoRNA depletion by RNAi in S2 cells

The plasmid *pValium20*(also known as *UAS-shRNA*) was used for the generation of short hairpin RNAs (shRNAs). Complementary oligonucleotides were annealed and ligated into *pValium20* [73] digested with *NheI* and *EcoRI*. The sequence of the oligonucleotides is given in Supplementary Table S1. The shRNA–snoRNA plasmids were transiently transfected into *SIN* replicon S2 cells along with pAc-GAL4 using standard methods, and the cells were harvested and analyzed 48 h after transfection. The sequence targeted by the shRNA is shown in Supplementary Fig. S1.

### Chromatin isolation by RNA purification in third instar larval brains

100 brains were dissected from *ActGAL4 > SIN* and *ActGAL4 > SINΔ* third instar larvae and chromatin isolation by RNA purification (ChIRP) was performed according to Chu *et al.* [74]. The biotin labeled anti-sense snoRNA probes used for pull down are listed in Supplementary Table S1. The purified RNA from the input and pull down samples was analyzed by quantitative reverse transcriptase-polymerase chain reaction to examine the specificity of the pull down using specific oligonucleotides. The purified DNA was analyzed by qPCR using oligonucleotides listed in Supplementary Table S1.

### Chromatin immunoprecipitation from larval brains

ChIP was performed as described by Botelho *et al.* [75] using the anti-rat Brg1 antibody [76] that recognizes the *Drosophila* Brahma protein. Briefly, 50 larval brains were used for each immunoprecipitation. The larval tissue was fixed in 2% formaldehyde followed by chromatin shearing to fragment sizes in the 200–900 bp range. Immunoprecipitation was performed with pre-cleared lysate overnight at 4°C using 10 μg/ml of anti-Brg1 antibody and Rabbit IgG as a negative control (Abcam, ab46540). A mix of Protein A and G Dynabeads (Invitrogen) blocked with 1 mg/ml BSA and 1 mg/ml salmon sperm DNA was used to capture antibody-chromatin complexes for 90 min, followed by four 5-min washes with radioimmunoprecipitation assay (RIPA) buffer containing 0.7% sodium deoxycholate. Further, the beads were washed once with 0.05 M Tris–HCl (pH 8.0), 2 mM EDTA. 10% of washed beads were reserved for RNA purification with TRIZOL (Invitrogen) after proteinase K treatment to reverse the crosslinking. The immunoprecipitated RNA was reverse transcribed and analyzed by RT-qPCR using standard protocols. The remaining 90% of the material was processed for DNA analysis. The crosslinking was reversed in Tris EDTA (TE) buffer containing 0.05% sodium dodecyl sulfate, 0.1 mg/ml RNase A, and 0.2 mg/ml proteinase K (Thermo Scientific) at 55°C for 3 h, and subsequently at 65°C overnight. The immunoprecipitated DNA was purified using the ChIP DNA Clean & Concentrator kit (ZymoResearch, D5205). For qPCR analysis, the KAPA SYBR Fast qPCR Kit (KAPA Biosystem) was used in BioMolecular Systems MIC instrument. The purified DNA was analyzed by qPCR using oligonucleotides listed in Supplementary Table S1.

### Pupation assays

50 first instar larvae from *SIN*, *Δ9B1;SIN*, *Δ9B3;SIN*, and *ΔA2486* with and without GAL4 were collected and distributed in fresh food vials. The number of larvae developing into pupae was monitored for 7 days. Percent pupation was calculated for each genotype relative to the number of pupae in controls without GAL4. Three biological replicates were performed, each with 50 animals for each genotype.

In another series of experiments, early second instar larvae were injected with 50 nl of 3.4e + 9 PFU/ml viral stock through the cuticle towards the posterior end. An injection of the same volume of DMEM (Gibco) supplemented with 10% FBS (Gibco) served as a mock-infected control. *w^1118^* larvae were used as background strain. Following injections, larvae were transferred to new vials and maintained at 25°C. They were monitored up to five days to follow pupation. Percentage of pupation was calculated for each genotype relative to the number of pupae in control samples. Three biological replicates were performed, each with 50 animals for each genotype.

### Statistical testing

In bar plots, the bars show average values and the error bars represent standard deviations unless otherwise indicated. The number of biological replicates for each experiment and the statistical tests used in each case are indicated in the figure legends. All plots and statistical analyses were performed using GraphPad Prism 10. Analyses of two sample means were performed using a two-tailed Student’s unpaired *t*-test, if the data passed normality test (Shapiro–Wilk test). Equal variances between the groups were ensured using an F-test (*P*>.05). For grouped data, one-way or two-way analysis of variance (ANOVA) combined with a two-stage linear step-up procedure of Benjamini, Krieger, and Yekutieli was applied. Probability values (p_adj_) for statistically significant differences are provided in the figures.

## Results

### Identification of ca-snoRNAs in *Drosophila* S2 cells

**Figure 1.**
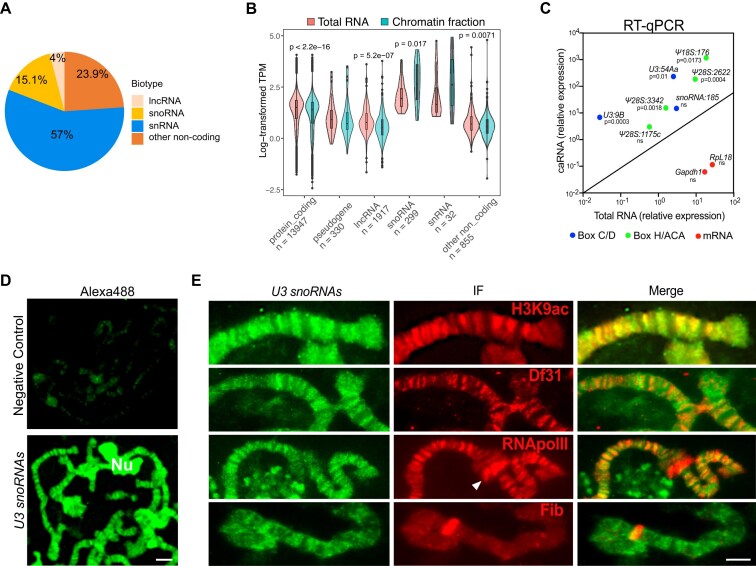
Identification of ca-snoRNAs in *D. melanogaster*. (**A**) Relative distribution of normalized counts per transcript for each noncoding RNA biotype, corresponding to the 28.7% of the total chromatin counts. Protein coding genes (71.3%) are not represented in the plot. (**B**) RNA abundances in total and chromatin RNA preparations normalized by length and sequencing depth (TPM). Nonparametric Wilcoxon rank test was used for statistical testing of RNA abundances. Counts have been log-transformed for visualization purposes. (**C**) Scatter plot showing the expression level of different ca-snoRNAs in total and chromatin fractions as measured by RT-qPCR. The *x*- and *y*-axes show relative RNA levels normalized to *Act5C*. The axes scales are log transformed. Box C/D snoRNAs and box H/ACA snoRNAs are represented by blue and green dots, respectively. Red dots represent mRNAs analyzed in parallel as examples of chromatin depleted transcripts. Two tailed nonparametric Mann–Whitney test was used to establish the statistical significance of the enrichment in chromatin compared to total RNA. N = 3. (**D**) RNA fluorescence *in situ*hybridization (RNA-FISH) using a DIG-labeled probe to show the distribution of *U3 snoRNAs* (lower panel) in polytene chromosomes of salivary glands of third instar larvae. The upper panel shows chromosomes hybridized in parallel with a negative control probe. Nu, nucleolus. The scale bar represents ∼25 μm. (**E**) RNA-FISH and immunofluorescence (IF) showing the co-localization of different proteins with *U3 snoRNA* in polytene chromosomes of salivary glands of third instar larvae. The names of the proteins tested are marked in each inset. The merged channel is shown in the right panel. Arrowhead indicates chromosome puff. The scale bar represents ∼10 μm.

We recently profiled caRNAs isolated from the chromatin fraction of S2 cells [56]. The analysis of a size-fractionated caRNA pool in the 0–500 nt range showed that snoRNAs constitute a large portion of the noncoding, chromatin-associated transcriptome (Fig. 1A). Additionally, analysis of the total chromatin fraction indicated that, despite considerable differences in the relative abundances of individual snoRNAs, snoRNAs as a biotype are significantly enriched in the chromatin (Fig. 1B). The chromatin-enriched snoRNAs are listed in Supplementary Table S2 and include both C/D box and H/ACA snoRNAs. The chromatin enrichment of selected snoRNAs was validated by RT-qPCR (Fig. 1C). An independent ChAR-seq study based on RNA–DNA proximity ligation identified a similar set of ca-snoRNAs in CME-W1-cl8+ *Drosophila* wing disc cells [3].

We chose to focus our study on *snoRNA:U3:9B* because it was one of the chromatin-enriched snoRNAs in the two studies referred to above and because ChAR-seq identified ∼140 000 DNA contacts for this snoRNA in the genome of *D. melanogaster*, which suggested a widespread role for *snoRNA:U3:9B* in the chromatin. A meta-analysis of the ChAR-seq data confirmed that *snoRNA:U3:9B* was highly enriched in the chromatin of wing disc cells (Supplementary Fig. S2A).

We carried out RNA-FISH on polytene chromosomes dissected from salivary glands of third instar larvae to validate the association of *snoRNA:U3:9B* with the chromatin. The DIG-labeled probe, complementary to a common region in the three *U3 snoRNA* paralogs of *D. melanogaster* (see Supplementary Fig. S1), localized to many loci in the polytene chromosome (Fig. 1D, bottom panel). The labeling was highly specific as shown by comparison to a DIG-labeled probe complementary to an unrelated bacterial sequence (Fig. 1D, upper panel). The previously reported association of snoRNAs with euchromatin [33] suggested that snoRNAs bind to transcriptionally active genomic regions. To test whether this was the case for *snoRNA:U3:9B*, we carried out double-labeling experiments combining RNA-FISH and IF with antibodies against marker proteins. The *U3 snoRNA* probe co-localized to a large extent with the active histone mark H3K9ac, with RNA polymerase II, and with Df31, a protein that was shown to be required for the maintenance of open chromatin [33] (Fig. 1E). Although widely distributed, the FISH signal in active loci was gene specific as revealed by the observation that certain chromosome puffs, which were intensely stained by the anti-RNA polymerase II antibody, were not labeled by the *U3 snoRNA* probe (white arrowhead in Fig. 1E).

Interestingly, double-labeling FISH experiments with an antibody against Fibrillarin showed a very low degree of colocalization (Fig. 1E, bottom panel), which suggests that *U3 snoRNAs* are associated to chromatin as part of a noncanonical snoRNP complexes that lack Fibrillarin.

The FISH probe used was capable of detecting all *U3 snoRNA* paralogues in *D. melanogaster*. To more precisely determine the association of *snoRNA:U3:9B* with open chromatin, we intersected the ChAR-seq data from Bell *et al.* [3] with available marker protein datasets. Interestingly, 90% of Df31 bound genes identified by Filion *et al.* [77] using DamID, and 95.8% of H3K9ac ChIP-seq peaks [3] overlapped with ChAR-seq contacts for *snoRNA:U3:9B* (Supplementary Fig. S2B). Together with the FISH results reported above, these observations strongly support the association of *snoRNA:U3:9B* with active chromatin.

Widespread localization in the polytene chromosomes and association with transcriptionally active chromatin were not unique to *snoRNA:U3:9B* and a DIG-labeled probe against another ca-snoRNA, *snoRNA:185*, also labeled many loci in the polytene chromosomes (Supplementary Fig. S2C and D).

In summary, we have identified ca-snoRNAs in *Drosophila* S2 cells and shown that *snoRNA:U3:9B* co-localizes with many transcriptionally active loci in the *Drosophila* genome.

### A meta-analysis of snoRNA binding sites links *snoRNA:U3:9B* to signaling pathways and to the immune response

**Figure 2.**
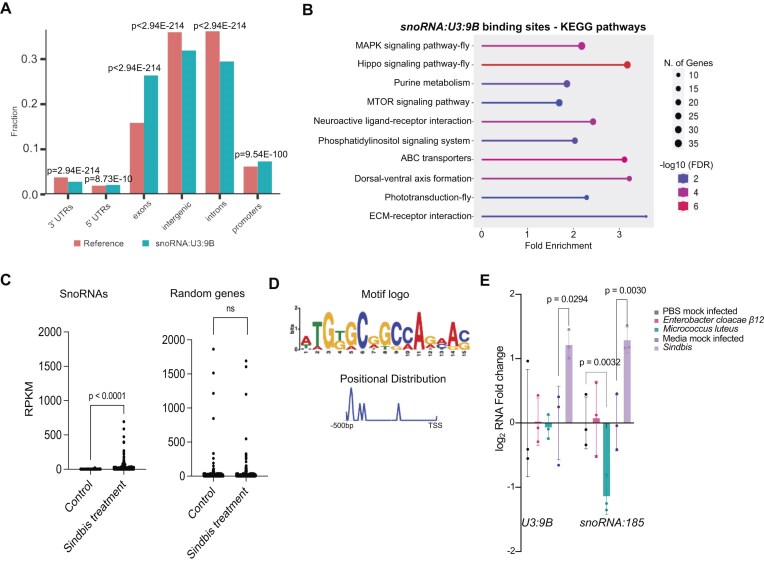
*SnoRNA:U3:9B* binds to protein-coding genes related to signaling pathways and is induced in response to viral infection. (**A**) Meta-analysis of ChAR-seq data [3]. The bar plot shows the association of *snoRNA:U3:9B* contact sites with specific gene features compared to the reference genome. Two-sample proportional z-test with Benjamini-Hochberg correction was used to assess the statistical significance of differences between the distributions. (**B**) Gene ontology enrichment analysis of Kyoto Encyclopedia of Genes and Genomes (KEGG) pathway of genes with at least 10 *snoRNA:U3:9B* binding sites according to ChAR-seq. The plot shows the top 10 KEGG pathways. Statistical significance is color-coded. The *x*-axis shows the fold enrichment of genes in each category. The size of the dot indicates the number of genes in each category. (**C**) Jitter plot showing the normalized expression of snoRNAs (n = 278) and a random set of genes (n = 278) in third instar larvae expressing the *SIN* replicon (Sindbis treatment) and in control animals not expressing the *SIN* replicon (Control, isogenic strain *y^1^; cn bw^1^sp^1^*). Data from the modENCODE project [78]. The *y*-axis shows normalized RPKMs as measured in modENCODE RNA-seq. Two-tailed unpaired Kolmogorov–Smirnov test was used to compare the data sets. (**D**) A total of 10 out of the 143 snoRNAs upregulated by the Sindbis treatment (see text for details) are expressed from independent genes (highlighted in red in Supplementary Table S2). The figure shows the sequence logo of the *lola-PO* binding motif identified by XSTREME [60] in these 10 genes (*E*-value = 5.95e-05, n = 10). The lower panel shows the position of the identified motif relative to the TSS. Two control sets of uninduced snoRNAs (including independent and polycistronic) or random protein coding genes were tested in parallel and no motif occurrence was identified in any of the sets (not shown). (**E**) Analysis of snoRNA expression in infected larvae. Bacteria or virus preparations were injected into third instar larvae, RNA was extracted from larval brains dissected 24 h after infection. SnoRNAs expression was analyzed by RT-qPCR and normalized to *Act5C*. Mock infected animals were analyzed in parallel. The RNA levels are expressed as log_2_ fold change compared to mock infected controls. Multiple comparisons were carried out using ordinary one-way ANOVA combined with a two-stage step-up procedure of Benjamini, Krieger, and Yekutieli. Adjusted *P*-values are shown in the figure. N = 3.

We mined the available ChAR-seq data [3] and classified the DNA contact points for *snoRNA:U3:9B* and *snoRNA:185* according to gene features. Both snoRNAs were preferentially enriched in exonic sequences and slightly underrepresented in intronic and intergenic sequences (Fig. 2A and Supplementary Fig. S3), which suggests that snoRNA recruitment to transcribed genes is at least partially mediated by the nascent mRNA.

According to the ChAR-seq data from Bell *et al.*[3], *snoRNA:U3:9B* makes 142 267 contacts in the *D. melanogaster* genome and is associated with 10 057 out of the 17 864 annotated genes. The number of contacts per gene ranged from 1 to 2460. Selection of genes with at least 10 contact points resulted in a list of 2492 genes which were the most preferred *snoRNA:U3:9B* bound genes (hereinafter referred to as *snoRNA:U3:9B* target genes). A gene ontology enrichment analysis of the 2492 *snoRNA:U3:9B* target genes revealed significant links to KEGG pathway terms MAPK/JNK signaling (enrichment FDR = 5.58e-05) and Hippo signaling (enrichment FDR = 1.53e-08) (Fig. 2B). These signaling pathways are involved in a variety of biological processes including the regulation of immune responses in mammals [53, 54]. The *snoRNA:U3:9B* target genes included 23 Toll pathway genes, and 16 Imd pathway genes (see Supplementary Table S3). These observations suggested a possible link between *snoRNA:U3:9B* and the immune responses of *D. melanogaster*. Interestingly, the analysis of publicly available data from the modENCODE gene expression project [78], which describes transcriptome changes in response to different experimental treatments, revealed that the expression of *snoRNA:U3:9B* was remarkably increased upon Sindbis virus treatment (Supplementary Table S2). The Sindbis virus treatment in modENCODE consists of expression of a noninfectious Sindbis replicon in a transgenic fly strain [72]. We compared the expression of each annotated snoRNA upon Sindbis treatment to its expression in untreated wild-type larvae (L3 puff stage 7–9). Interestingly, not only *snoRNA:U3:9B* but as many as 144 out of the 278 annotated snoRNA genes were induced by the Sindbis treatment in third instar larvae (Fig. 2C). Out of these 144 snoRNAs, 105 were found to be chromatin associated in our caRNA analysis (Supplementary Table S2).

We further investigated whether there was any underlying regulatory feature in the TSS of Sindbis-induced snoRNAs. Out of 144 induced snoRNAs, only 10 are transcribed from independent genes. The remaining 134 are derived from either introns or polycistronic host genes, with only two of these showing upregulation upon Sindbis treatment. Thus we searched for known motifs upstream of the TSS of the 10 independent snoRNA genes that were induced by the Sindbis treatment (highlighted in Supplementary Table S2). As compared to a control set of uninduced snoRNAs or random protein coding genes, the set of 10 virus-induced snoRNAs harbored binding motifs for the transcription factor *lola* (longitudinals lacking, *lola-PO*) (Fig. 2D; *E*-value = 5.95e-05). The *lola* motif was identified in nine out of ten snoRNAs and was located in a window 400–500 bp upstream of the TSS (Fig. 2D). Previous studies have shown that *lola* is implicated in the regulation of immune signaling pathways [79, 80].

To investigate whether snoRNA induction is a general response to immune challenges in *Drosophila*, we performed different microbial infections in early third instar larvae and quantified the expression of two ca-snoRNAs in larval brain 24 h post infection. The levels of *snoRNA:U3:9B* and *snoRNA:185* increased significantly following Sindbis virus infection (Fig. 2E), but were not affected by infection with Gram-negative *Enterobacter cloacae* β12 or Gram-positive *M. luteus*. Infection with a VSV also resulted in *snoRNA:U3:9B* and *snoRNA:185* upregulation (Supplementary Fig. S4). These results linked ca-snoRNAs to viral infection. The modest increase in the expression of these *snoRNAs* suggests that they are unlikely to function as antiviral effectors. Rather, *snoRNA:U3:9B* and *snoRNA:185* may play a regulatory role in the antiviral immune response.

The association of ca-snoRNAs with the chromatin and with signaling pathway genes in particular, and the induction of snoRNAs following a viral infection were interesting observations that suggested a function for some snoRNAs in regulating chromatin changes related to antiviral responses.

### The Sindbis replicon as a model to study the activation of immune response genes

**Figure 3.**
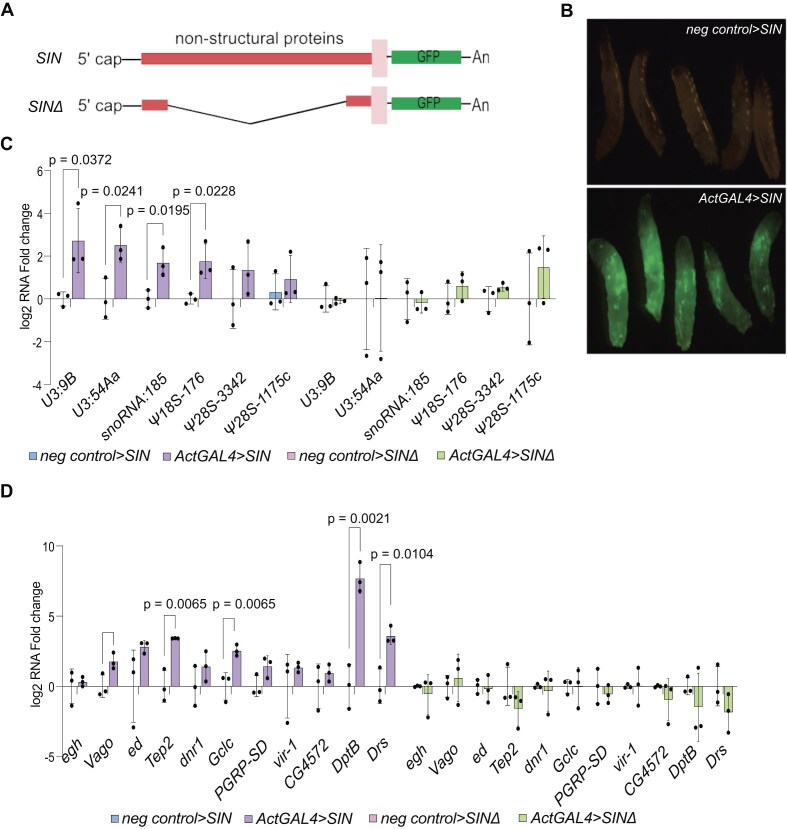
*SIN* replicon expression induces the expression of immune response genes in third instar larvae. (**A**) Schematic representation of the Sindbis replicon model. *SIN* encodes the 5′ cap, four nonstructural proteins, and a GFP open reading frame under the control of the sub-genomic promoter. *SINΔ*carries a deletion of nonstructural protein sequences. The replicon is expressed under the control of the UAS/GAL4 system. (**B**) Representative fluorescence images of third instar larvae showing the expression of GFP in control *SIN* (upper) and *ActGAL4 > SIN* (lower). (**C**) Bar plot showing the induction of ca-snoRNA expression in dissected brains of *SIN* third instar larvae with and without *ActGAL4* driver. The graph also shows the lack of induction of ca-snoRNAs in *SINΔ* larval brains with and without *Act*GAL4. The expression was measured by RT-qPCR and normalized to *Act5C*. The RNA levels are expressed as log_2_ fold change compared to the respective controls. Two tailed Student’s unpaired *t*-test was used to compare the data sets. N = 3. (**D**) Bar plot showing the induction of target immune response genes in the same four conditions described in panel (C). The expression was measured by RT-qPCR and normalized to *Act5C*. The RNA levels are expressed as log_2_ fold change compared to controls. Two tailed Student’s unpaired *t*-test was used to compare the data sets. N = 3.

We used the Sindbis infection model developed by Avadhanula *et al.* [72] to study the function of *snoRNA:U3:9B* and its impact on the expression of antiviral genes. This Sindbis infection model utilizes an engineered Sindbis replicon in which the structural protein genes have been replaced by a GFP cassette (Fig. 3A). The resulting replicon has the potential to replicate but fails to produce infectious particles. The fly strain carrying this Sindbis-GFP replicon, initially called SINrep:GFP, is hereafter referred to as “*SIN*” for short. A related strain, SINΔrep:GFP (hereafter referred to as “*SINΔ*”), carries a deleted Sindbis replicon that lacks most of the nonstructural protein coding sequences *nSP1-4*. In these strains, the expression of the Sindbis replicon was induced by crossing *SIN* (or *SINΔ* as a control) with flies that express the GAL4 driver under the control of the *Act5C* promoter (*ActGAL4*). A representative image of *SIN* third instar larvae expressing the Sindbis replicon driven by *ActGAL4* and traced by GFP expression is shown in Fig. 3B. The control driverless larvae are hereafter referred to as *neg control > SIN*.

The level of expression of different ca-snoRNAs was measured in two organs from third instar larvae: brain because the Sindbis virus is known to infect neurons [81] and cardia because of the prominent role of the gut in the *Drosophila* immune response [82]. GAL4-driven expression of the *SIN* replicon, but not expression of the *SINΔ* replicon, resulted in significant upregulation of several ca-snoRNAs in both brain and cardia (Fig. 3C and Supplementary Fig. S5A, respectively), in agreement with the modENCODE data reported in Fig. 2C. Neither *snoRNA:U3:9B* nor *snoRNA:185* were induced in *ActGAL4 > SINΔ* larvae, which directly links the increased expression of selected snoRNAs to the activation of the *SIN* replicon.

We also asked whether the activation of the *SIN* replicon would induce the expression of antiviral response genes. We analyzed by RT-qPCR the relative expression of transcripts from some *snoRNA:U3:9B* target genes identified by ChAR-seq that were ascribed to the immune response pathway. Larvae expressing the *SIN* replicon (*ActGAL4 > SIN* larvae), but not *ActGAL4 > SINΔ* larvae, showed increased expression of selected immune response genes in brain and cardia, as shown in Fig. 3D and Supplementary Fig. S5B, respectively. Specifically, genes such as *Vago* [47], *DptB* [83] or *Tep2* [84] that are known to be induced upon Sindbis virus infection were significantly upregulated in our infection model. In summary, these results showed that expression of the *SIN* replicon in third instar larvae resulted in increased expression of a subset ca-snoRNAs and target immune response genes.

### *SnoRNA:U3:9B* is required for the activation of immune response genes

We created two independent *snoRNA:U3:9B* knockout fly strains using standard gRNA mediated CRISPR-Cas9 technology to investigate the functional importance of *snoRNA:U3:9B in vivo* (Supplementary Fig. S6A). The specificity of the deletions was confirmed by PCR analysis of single fly genomic DNA (Supplementary Fig. S6B). The two deletion lines, Δ*9B1* and Δ*9B3*, were viable. They were crossed with *SIN* flies to create *snoRNA:U3:9B* knockout *SIN* flies (Δ*9B1;SIN and* Δ*9B3;SIN*). In all cases, the expression of the *SIN* replicon was driven by *ActGAL4*. RT-qPCR analysis of *nSP1* and *GFP* expression showed that the *SIN* replicon was expressed at similar levels in wild-type, *snoRNA:U3:9B* deletion strains, and a control snoRNA deletion Δ*A2486* strain (Supplementary Fig. S6C). The snoRNA levels were also measured in these backgrounds to confirm the snoRNA deletions in the knockout strains as well as the induction of *snoRNA:U3:9B* expression in the wild-type strains (Supplementary Fig. S6D). *SnoRNA:U3:9B* was undetectable in the Δ*9B1* and Δ*9B3* flies, and significantly induced in both wild-type or Δ*A2486* knockout on *ActGal4*-driven *SIN* replicon expression (Supplementary Fig. S6D). The levels of *snoRNA:U3:54Aa/b* and *snoRNA:185* remained unchanged in the deletion strains.

**Figure 4.**
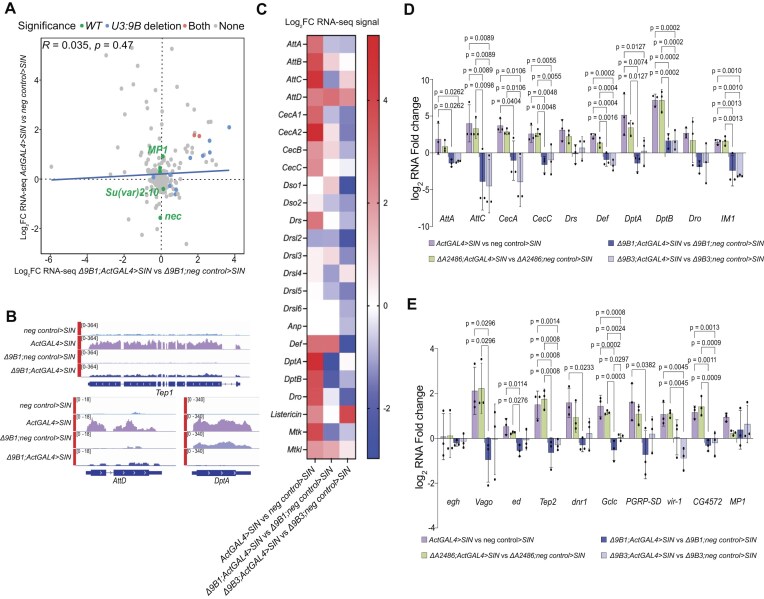
*SnoRNA:U3:9B* deletion inhibits the activation of target immune response genes in response to *SIN* replicon expression. (**A**) Scatter plot comparing the transcriptional immune response to *SIN* expression in *snoRNA:U3:9B* knock-out (*Δ9B1; ActGAL4 > SIN* versus *Δ9B1; neg control > SIN*, *x*-axis) and wild-type (*ActGAL4 > SIN* versus *neg control > SIN*, *y*-axis) larval brains. In each strain, changes in normalized RNA levels for transcripts categorized under GO:immune system process (GO:0 002 376) with and without GAL4 were expressed as log_2_ fold change (log_2_FC). The plot shows data from three independent biological replicates. Transcripts in green were differentially expressed (p_adj_< 0.05) in response to *SIN* replicon expression only in wild-type (*SIN*), blue transcripts only in *snoRNA:U3:9B* knockout (*Δ9B1; SIN)*, and red transcripts in both conditions. Two transcripts that were significantly changed only in *Δ9B1; ActGAL4 > SIN* versus *Δ9B1; neg control > SIN*, namely *IM1* (log_2_FC = −16.11017754, p_adj_= 2.95e-08) and *mRpL53* (log_2_FC = −23.45474574, p_adj_= 9.12e-04), showed extreme values and were removed from the scatter plot for representation purposes. (**B**) Representative genome browser screenshots of RNA-seq signal in wild-type (*SIN*) and *snoRNA:U3:9B* knock out (*Δ9B1;SIN*) with and without expression of *SIN* replicon. The signal tracks represent coverage of normalized reads from three independent biological replicates across the genome. The thick blue boxes represent exons, lines represent introns, and the arrowheads indicate the gene direction. (**C**) Heat map showing AMP gene expression changes in brains of wild-type (*ActGAL4 > SIN* versus *neg control > SIN*) or *snoRNA:U3:9B* knock-out larvae (*Δ9B1; ActGAL4 > SIN* versus *Δ9B1; neg control > SIN* and *Δ9B3; ActGAL4 > SIN* versus *Δ9B3; neg control > SIN*) in response to *SIN* replicon expression. Log_2_FC reflects mean value comparing with and without GAL4 from three independent biological replicates. (**D**) Bar plot showing the activation of AMP genes in larval brains in response to *SIN* replicon expression. Expression levels were quantified in brains from *SIN* replicon expressing third instar larvae in wild-type (*ActGAL4 > SIN* versus *neg control > SIN*), control snoRNA knock-out (*ΔA2486; ActGAL4 > SIN* versus *ΔA2486; neg control > SIN*) or *snoRNA:U3:9B* knock-out lines (*Δ9B1; ActGAL4 > SIN* versus *Δ9B1; neg control > SIN* and *Δ9B3; ActGAL4 > SIN* versus *Δ9B3; neg control > SIN*) by RT-qPCR, normalized to *Act5C* and expressed as log_2_FC relative to normalized expression in brains without GAL4. Multiple comparisons were carried out using ordinary one-way ANOVA combined with a two-stage step-up procedure of Benjamini, Krieger, and Yekutieli. Adjusted *P*-values are shown in the figure. N = 3. (**E**) Bar plot showing the expression of selected *snoRNA:U3:9B* target immune response genes in larval brains in response to *SIN* replicon expression. Expression levels were measured by RT-qPCR as in panel (D). N = 3.

We profiled the brain transcriptome of wild-type and *snoRNA:U3:9B* knockout larvae, with and without *SIN* replicon expression (Supplementary Fig. S7). A principal component analysis indicated that deletion of *snoRNA:U3:9B* did not cause major changes in the transcriptome of infected larval brain (Supplementary Fig. S7A). The targeted analysis of 489 genes annotated as immune response genes (GO:0002376) did not reveal global effects of *snoRNA:U3:9B* deletion on the immune response, but identified a few immune response genes that were differentially expressed in response to *SIN* replicon expression in wild-type but not in *snoRNA:U3:9B* knockout larvae (Fig. 4A). For instance, *MP1*, a gene that encodes a serine proteases that cleaves Spätzle and activates the Toll pathway [85], was significantly upregulated upon expression of the *SIN* replicon in wildtype conditions but not in *snoRNA:U3:9B* knockout larvae (Fig. 4A and E). Interestingly, many AMP genes that were normally upregulated upon expression of the *ActGAL4 > SIN* replicon in wildtype larvae were unchanged or showed decreased expression in Δ*9B1; ActGAL4 > SIN* and Δ*9B3; ActGAL4 > SIN* larvae (Fig. 4B and C). In many cases, the differences in AMP gene expression observed in the RNA-seq analysis were not statistically significant due to variability in the AMP gene activation levels. Although the cause of this variation remains unclear, it may be related to the use of *ActGAL4*-mediated activation of the SIN replicon, which starts already in embryos and could trigger secondary effects, thereby contributing to variability in the magnitude of the response. In spite of this variability, the differences were reproducible throughout replicates and could be validated by RT-qPCR (Fig. 4D). RT-qPCR also demonstrated significant defects in the induction of other innate immune genes such as *Vago*, *PGRP-SD* and *vir-1* that were not identified by the differential expression analysis due to large variability in the extent of the responses (Fig. 4E). In summary, the loss-of-function experiments presented above showed that depletion of *snoRNA:U3:9B* does not cause widespread disruption of the transcriptome. Instead, it specifically inhibits the transcriptional activation of a subset of immune response genes, which demonstrates a role for *snoRNA:U3:9B* in the immune response to Sindbis virus.

### Changes in chromatin accessibility related to the activation of immune response genes require *snoRNA:U3:9B*

A previous study on a possible role for snoRNAs in maintaining open chromatin states [33] and the fact that *snoRNA:U3:9B* was associated with chromatin [3, 56] prompted us to study whether the effect of *snoRNA:U3:9B* on the expression of immune response genes entailed changes in chromatin accessibility. We carried out ATAC-seq experiments in wild-type and *snoRNA:U3:9B* knockout larvae upon GAL4-driven expression of the *SIN* replicon (Supplementary Fig. S8A).

A differential accessibility analysis revealed that *snoRNA:U3:9B* deletion resulted in severe chromatin changes in the absence of any immune challenge. A total of 1376 differentially accessible regions were detected in Δ*9B1; neg control > SIN* and Δ*9B3; neg control > SIN* larvae compared to wild-type. These regions were located at or near 978 genes. A gene ontology enrichment analysis revealed that the affected genes were linked to developmental and morphogenetic processes (Supplementary Fig. S8B and C), which suggests that *snoRNA:U3:9B* is required to maintain the chromatin homeostasis of regulated genes.

**Figure 5.**
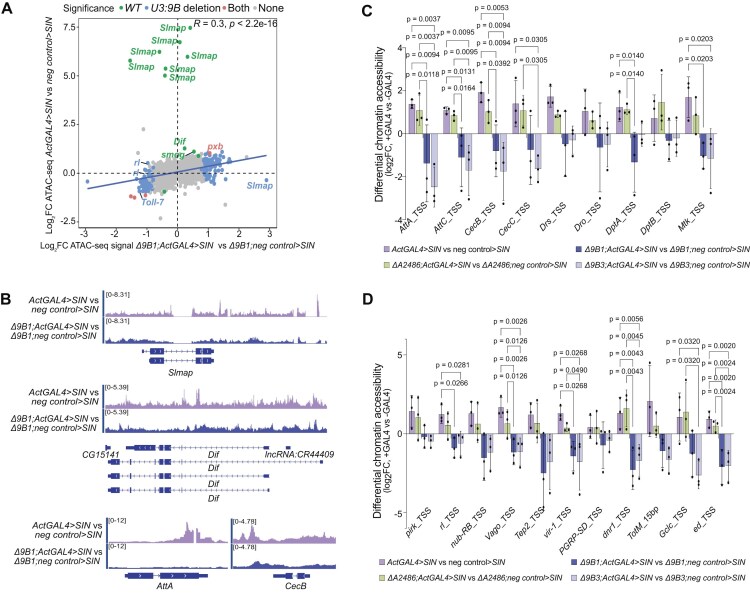
*SnoRNA:U3:9B* regulates the chromatin accessibility of immune response genes in *SIN* replicon expressing larvae. (**A**) Scatter plot showing differentially accessible regions (as measured by ATAC-seq) in genes categorized under GO:immune system process (GO:0002376) in larval brains in *snoRNA:U3:9B* knock-out (*Δ9B1; ActGAL4 > SIN* versus *Δ9B1; neg control > SIN*, *x*-axis) and wild-type (*ActGAL4 > SIN* versus *neg control > SIN*, *y*-axis). Accessibility changes are expressed as log_2_FC comparing with and without GAL4 from three independent biological replicates. Regions in green were differentially accessible (p_adj_< 0.05) in response to *SIN* replicon expression only in wild-type (*ActGAL4 > SIN* versus *neg control > SIN*), blue dots represent regions that were significant only in *snoRNA:U3:9B* knockout (*Δ9B1; ActGAL4 > SIN* versus *Δ9B1; neg control > SIN)*, and red regions showed significant changes in both conditions. (**B**) Representative genome browser screenshots showing changes in ATAC-seq signal in wild-type (*ActGAL4 > SIN* versus *neg control > SIN*) and *snoRNA:U3:9B* knock out (*Δ9B1; ActGAL4 > SIN* versus *Δ9B1; neg control > SIN*) with and without expression of *SIN* replicon. The signal tracks show ratios of normalized ATAC-seq signals. (**C**) Bar plots showing differentially accessible regions at AMP genes in brains of *SIN* replicon expressing third instar larvae in wild-type (*ActGAL4 > SIN* versus *neg control > SIN*), control snoRNA knock-out (*ΔA2486; ActGAL4 > SIN* versus *ΔA2486; neg control > SIN*) or *snoRNA:U3:9B* knock-out (*Δ9B1; ActGAL4 > SIN* versus *Δ9B1; neg control > SIN* and (*Δ9B3; ActGAL4 > SIN* versus *Δ9B3; neg control > SIN*). The accessibility was measured by ATAC-qPCR, normalized to a silent heterochromatic region in chr2R, and expressed as log_2_FC relative to control brains without GAL4. The primer sequences and genomic coordinates for the analyzed regions are provided in Supplementary Table S1. Multiple comparisons were carried out using ordinary one-way ANOVA combined with a two-stage step-up procedure of Benjamini, Krieger, and Yekutieli. Adjusted *P*-values are shown in the figure. N = 3. (**D**) Bar plots showing differentially accessible regions in genes that code for immune regulators analyzed by ATAC-qPCR as in panel (**C**). N = 3.

The ATAC-seq analysis of *ActGAL4 > SIN* larvae identified 297 differentially accessible regions located at or near 201 genes in *snoRNA:U3:9B* knockout compared to wild-type (Supplementary Fig. S9). The targeted analysis of accessibility changes in the 489 genes annotated as immune response genes (GO:0002376) revealed changed accessibility in several genes that code for immune regulators. For example, multiple regions with increased accessibility in wild-type but not in *snoRNA:U3:9B* knockout larvae were identified at/near the *Slmap* gene, which codes for a negative regulator of the Hippo and Imd pathways [86]. Other affected genes were *Dif*, which encodes a NF-κB type transcription factor that regulates AMP expression through the Toll pathway [87] and *Toll-7*, which induces antiviral autophagy [88] (Fig. 5A and B).

ATAC-qPCR revealed that chromatin accessibility at AMP genes was normally increased upon infection in wild-type larvae (*ActGAL4 > SIN*) but not in *snoRNA:U3:9B* knockout larvae (*Δ9B1; ActGAL4 > SIN* and *Δ9B3; ActGAL4 > SIN*) (Fig. 5B and C). Moreover, the defects in the induction of genes previously linked to Sindbis virus infection (such as *Vago*, *vir-1* or *ed*) or involved in the Imd pathway [such as *pirk*, *rolled (rl)/Erk*, *nub*, or *dnr1*] reported in Fig. 4E were in most cases also accompanied by defects in chromatin accessibility (Fig. 5D). These observations indicate that *snoRNAU3:9B* is required for the chromatin rearrangements that are associated with the activation of the antiviral response.

In a parallel series of experiments, we studied the involvement of *snoRNA:U3:9B* in immune activation in S2 cells. To this end, we established stable S2 cell lines by transfection of either pSIN or pSINΔ constructs, and induced *SIN* replicon expression in the stably transfected S2 cells by transient transfection of a pAc-GAL4 plasmid. *SIN* replicon expression resulted in *snoRNA:U3:9B* upregulation and induction of immune response genes, as in larval tissues (Supplementary Fig. S10A–C)). Moreover, the induction of immune response genes was correlated with an increase in chromatin accessibility, as demonstrated by ATAC-qPCR (Supplementary Fig. S10D). Notably, the depletion of *snoRNA:U3:9B* by RNA interference effectively counteracted this effect while depletion of an unrelated snoRNA did not (Supplementary Fig. S10E–G)).

Collectively, these findings revealed the importance of *snoRNA:U3:9B* in the regulation of antiviral gene expression and highlight its essential role in the chromatin accessibility changes that take place at specific gene loci in response to viral infection.

### *SnoRNA:U3:9B* binds to chromatin and promotes the association of Brahma to immune response genes in response to viral infection

**Figure 6.**
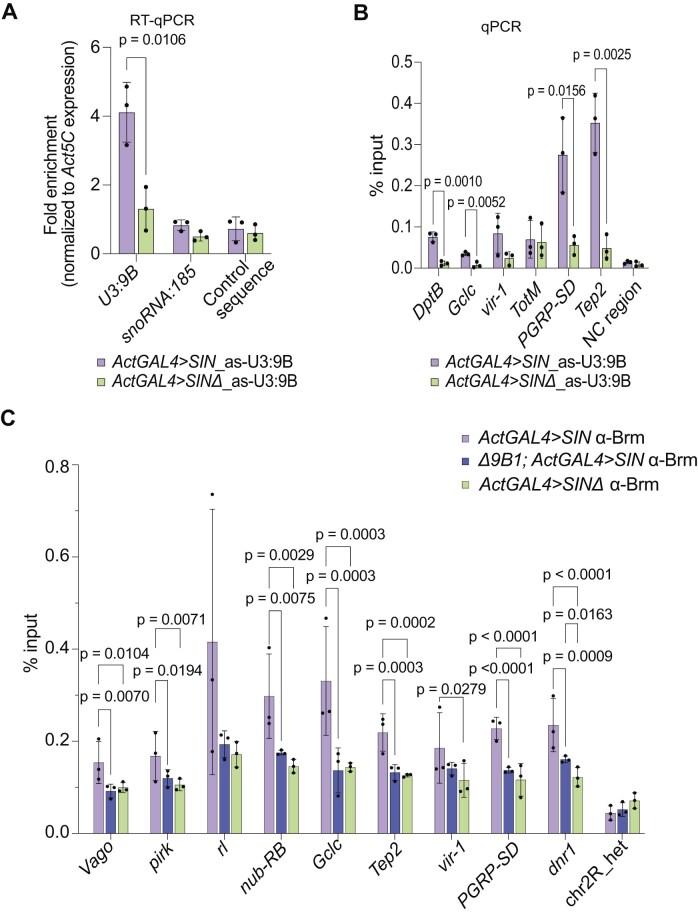
*SnoRNA:U3:9B* is required for the association of Brahma with target immune response genes. (**A**) Bar plot showing ChIRP specificity. *SnoRNA:U3:9B*, *snoRNA:185* and a control unrelated sequence were quantified by RT-qPCR in RNA samples isolated from larval brain tissue from *ActGAL4 > SIN* and *ActGAL4 > SINΔ* by ChIRP using biotinylated DNA probes complementary to *snoRNA:U3:9B* (as-U3:9B). Two tailed Student’s unpaired *t*-test was used to compare the data sets. N = 3. (**B**) Bar plot showing the association of *snoRNA:U3:9B* with specific target genes. DNA isolated by ChIRP was analyzed by qPCR using primers for the indicated genes. ChIRP was performed using chromatin from dissected larval brain samples from *ActGAL4 > SIN* and *ActGAL4 > SINΔ* , as indicated. Two tailed Student’s unpaired *t*-test was used to compare the data sets. N = 3. (**C**) ChIP-qPCR to analyze the presence of Brahma at the TSS of immune response genes. ChIP was performed in third instar larval brains from wild-type (*ActGAL4 > SIN*), *snoRNA:U3:9B* knockout (*Δ9B1; ActGAL4 > SIN*) and control *ActGAL4 > SINΔ* lines. An IgG antibody was used in parallel as a negative control. Multiple comparisons were carried out using ordinary one-way ANOVA combined with a two-stage step-up procedure of Benjamini, Krieger, and Yekutieli. Adjusted *P*-values are shown in the figure. N = 3.

To elucidate the mechanistic relationship between *snoRNA:U3:9B* and the observed alterations in chromatin accessibility, we investigated the physical association between *snoRNA:U3:9B* and antiviral response genes. To this end, we performed ChIRP using fixed dissected larval brains from *ActGAL4 > SIN* and *ActGAL4 > SINΔ* larvae. In an initial series of experiments, we investigated the bound RNAs using RT-qPCR. Antisense probes specific for *snoRNA:U3:9B* (as-U3:9B; Fig. 6A) or *snoRNA:185* (as-snoRNA:185; Supplementary Fig. S11A) effectively pulled down their respective snoRNAs, with enhanced yields observed in *ActGAL4 > SIN* compared to *ActGAL4 > SINΔ*. The observed difference in pulldown yield could be attributed, at least in part, to the increased expression levels of snoRNAs in cells expressing the *SIN* replicon. Both probes failed to pull down a control unrelated sequence (Fig. 6A and Supplementary Fig. S11A), which supports the specificity of the experiment, and the primers used for the RT-qPCR reactions did not amplify the probe itself (data not shown).

We subsequently analyzed the bound DNA by qPCR using primers specific to several genes previously linked to the *Drosophila* antiviral response. Our analysis revealed an association between *snoRNA:U3:9B* and target immune response genes, with this association being significantly stronger in the *ActGAL4 > SIN* larval brains than in those of *ActGAL4 > SINΔ* larvae (Fig. 6B). The as-snoRNA:185 probe also pulled down detectable amounts of DNA, but the enrichment in *ActGAL4 > SIN* compared to *ActGAL4 > SINΔ* was not significant (Supplementary Fig. S11B).

The ChIRP results presented above suggested that *snoRNA:U3:9B* acts locally in the chromatin, but the molecular interactions that mediate the association of ca-snoRNAs with chromatin are not understood. Based on the canonical mechanism of snoRNA action, we hypothesized that *snoRNA:U3:9B* forms base-pairing interactions with nascent pre-mRNAs at specific gene loci. To investigate this possibility, we used the snoRNA target prediction tool snoGloBe to identify potential RNA:RNA interactions that could explain the binding of *snoRNA:U3:9B* to its target genes. SnoGloBe predicted the interaction of *snoRNA:U3:9B* with 5S rRNA loci on chromosome 2R (Supplementary Fig. S12A, highlighted in orange) through a sequence that overlaps with the predicted canonical 3′ hinge (red line). SnoGloBe also predicted a large number of possible mRNA interaction partners for *snoRNA:U3:9B* in the transcriptome (n = 2857 genes with >5 interaction sites), including many immune response genes (Supplementary Fig. S10B and C). The predicted interactions mapped to both exons and introns (Supplementary Table S4), and a gene ontology (GO) analysis of the genes predicted to interact with *snoRNA:U3:9B* revealed a significant enrichment of signaling pathways that were very similar to those obtained for the *snoRNA:U3:9B* target genes identified by ChAR-seq, including the MAPK/JNK and Hippo pathways (compare Supplementary Fig. S10D with Fig. 2B).

*SnoRNA:U3:9B* is almost identical to two other snoRNAs encoded in the genome of *D. melanogaster*, *snoRNA:U3:54Aa* and *snoRNA:U3:54Ab*, but has a unique stretch of 37 nt (Supplementary Fig. S10A; nt 126–162 highlighted in green). Using a stringent *P*-value cutoff (*P*< 0.98), snoGloBe predicted 17 733 interaction sites for *snoRNA:U3:9B* in the transcriptome of *D. melanogaster*. Of these 17 733 sites, 893 sites mapped to 266 immune response genes. Among them we found the *Toll-7* receptor, the MAP kinase *rolled (rl)/Erk* and the transcription factor *nub* (Supplementary Table S5). A motif search in the 17 733 target sequences predicted by snoGlobe identified three highly significant motifs (Supplementary Fig. S12A and Supplementary Table S6). Motif 1 (GCCCCGCUCCCCG) and motif 3 (UGCAGUGCAUCC), which are complementary to sequences in the unique *snoRNA:U3:9B* stretch (Supplementary Fig. S12A), were found in 122 and 39 immune response genes, respectively (Supplementary Table S5). SnoGlobe also predicted interactions involving sequences that are common to the three *U3 snoRNAs* (not shown) and the biological significance of these interactions remains to be investigated. In summary, the *snoRNA:U3:9B*-specific interactions predicted by snoGloBe in many immune genes, the identification of highly significant sequence motifs and the GO similarities among bound and predicted genes (identified by ChAR-seq and snoGloBe, respectively) support the proposal that direct snoRNA:pre-mRNA base-pairing interactions contribute to the targeting of *snoRNA:U3:9B* to specific loci in the chromatin.

We also investigated a possible mechanism through which *snoRNA:U3:9B* could influence chromatin accessibility. Previous studies have shown that SWI/SNF chromatin remodeling complexes regulate the expression of immune genes in mammals [89] and *Drosophila* [90] and, in some cases, SWI/SNF does so in cooperation with RNAs [91]. In insects, the SWI/SNF catalytic subunit Brahma associates with nascent pre-mRNAs [92], and the profiling of the SWI/SNF-RNA interactome in human cells has revealed that transcriptional activation driven by SWI/SNF often entails SWI/SNF-RNA interactions [93]. Based on these earlier observations, we hypothesized that *snoRNA:U3:9B* was involved in the recruitment of SWI/SNF to AMP genes. We prepared chromatin from larval brains, carried out ChIP with an anti-Brahma antibody, and analyzed the immunoprecipitated RNA by RT-qPCR. Interestingly, *snoRNA:U3:9B* was specifically pulled down, which shows that Brahma and *snoRNA:U3:9B* are present in the same chromatin environment (Supplementary Fig. S13A). Moreover, using ChIP-qPCR, we detected Brahma associated with AMP genes and with other immune genes including regulators of innate immunity such as *rolled (rl)/Erk* [94] *and nub-RB* [95] (Supplementary Fig. 13B and C and Fig. 6C). In most cases, Brahma occupancy was significantly increased in immune-challenged wild-type larvae (*ActGAL4 > SIN*) compared to *ActGAL4 > SINΔ* controls. Importantly, this increase was completely abolished in *Δ9B1; ActGAL4 > SIN* larvae for several of the analyzed genes such as *Vago*, *rl*, and *nub-RB* that encode key regulators of immune gene expression (Fig. 6C and Supplementary Fig. S13C). However, the deletion of *snoRNA:U3:9B* had only a modest effect on the recruitment of Brahma to AMP genes (Supplementary Fig. S13B). These results indicated a critical role for *snoRNA:U3:9B* in the Brahma-mediated activation of a few specific immune regulators, which is required for the subsequent activation of immune effectors such as AMPs.

Altogether, the results of the ChIRP experiments suggest that *snoRNA:U3:9B* is physically associated with the chromatin of a specific group of immune response genes, that this association is mediated by RNA:RNA interactions, and that *snoRNA:U3:9B* acts locally to facilitate the recruitment of Brahma and an open chromatin state in response to Sindbis virus infection.

### *SnoRNA:U3:9B* is essential to survive immune stress

**Figure 7.**
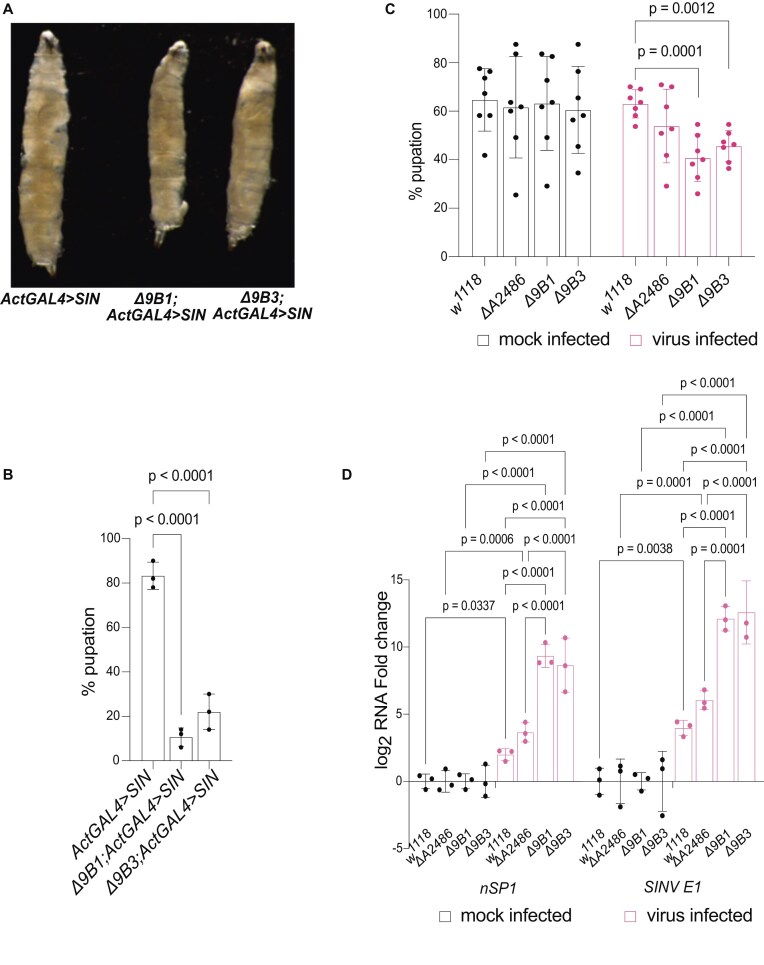
Knockout of *snoRNA:U3:9B* leads to reduced survival rates following a viral challenge. (**A**) Bright field images of representative wild-type (*ActGAL4 > SIN*) and *snoRNA:U3:9B* knock out (*Δ9B1; ActGAL4 > SIN* and *Δ9B3; ActGAL4 > SIN*) larvae expressing the *SIN* replicon under the control of the *ActGAL4* driver. (**B**) Bar plot showing the percentage of larvae pupating in a control line (*ActGAL4 > SIN*) and two *snoRNA:U3:9B* deletion lines (*Δ9B1; ActGAL4 > SIN* and *Δ9B3; ActGAL4 > SIN*). Multiple comparisons were carried out using ordinary one-way ANOVA combined with a two-stage step-up procedure of Benjamini, Krieger, and Yekutieli. Adjusted *P*-values are shown in the figure. N = 3 with 50 animals in each replicate and group. (**C**) Bar plot showing the percentage of larvae pupating in a control line (*w^1118^*), control snoRNA deletion line (*ΔA2486*) and two *snoRNA:U3:9B* deletion lines (*Δ9B1* and *Δ9B3*), either mock infected or infected with a Sindbis virus. Multiple comparisons were carried out using two-way ANOVA combined with a two-stage linear step-up procedure of Benjamini, Krieger, and Yekutieli. Adjusted *P*-values are shown. N = 7 independent experiments, each with 50 animals injected for each genotype. (**D**) Bar plot showing the relative levels of two viral transcripts, *nSP1* and *SINV E1*, in larval brains of third instar larvae in a control line (*w^1118^*), control snoRNA deletion line (*ΔA2486*), and two *snoRNA:U3:9B* deletion lines (*Δ9B1* and *Δ9B3*), either mock infected or infected with a Sindbis virus. Multiple comparisons were carried out using two-way ANOVA combined with a two-stage linear step-up procedure of Benjamini, Krieger, and Yekutieli. Adjusted *P*-values are shown. N = 3.

Next, we investigated whether the role of *snoRNA:U3:9B* in the chromatin and its involvement in the activation of immune response genes was physiologically relevant *in vivo*. The analysis of the phenotype of *SIN* replicon expressing flies revealed that *Δ9B1; ActGAL4 > SIN* and *Δ9B3; ActGAL4 > SIN* larvae were smaller than control *ActGAL4 > SIN* larvae (Fig. 7A). Scoring pupation frequencies, the pupation percentage was above 80% in the wild-type *ActGAL4 > SIN* strain, but only 10% and 22% in *Δ9B1; ActGAL4 > SIN* and *Δ9B3; ActGAL4 > SIN*, respectively (Fig. 7B). Interestingly, most *Δ9B1; ActGAL4 > SIN* and *Δ9B3; ActGAL4 > SIN* larvae died as feeding third instar larvae and never transitioned to wandering third instar larvae, which indicated that *snoRNA:U3:9B* is essential to survive the stress introduced by expression of the *SIN* replicon in third instar larvae. To further establish the physiological importance of *snoRNA:U3:9B*, we injected Sindbis virus into the hemolymph of third instar larvae and analyzed pupation frequencies as above. In mock infection experiments, the pupation frequency was ∼60% regardless of the genotype (Fig. 7C). When Sindbis virus was injected into wild-type *w^1118^* larvae, the frequency was again ∼60%, as in mock-infected wild-type animals. However, the pupation frequency was only 40.7% and 45.6%, respectively, for *Δ9B1* and *Δ9B3* infected larvae. Deletion of another snoRNA, *snoRNA:Me28S-A2486*, did not result in significant reduction of pupation frequency (53.8%; Fig. 7C). Further, we checked the levels of viral transcripts, *nSP1* and capsid protein coding gene, *SINV E1* by RT-qPCR in the different fly strains with and without Sindbis virus infection (Fig. 7D). The levels of viral transcripts were virtually undetectable in mock-infected animals, as expected. Following infection, the levels of *nSP1* and *SINV E1* were significantly higher in the *snoRNA:U3:9B* knockout strains than in the wild-type or control snoRNA deletion. The significant reduction of pupation frequencies and increased viral replication observed in *snoRNA:U3:9B* knockout larvae demonstrated that *snoRNA:U3:9B* is specifically required for efficient antiviral defence *in vivo*.

## Discussion

RNA has long been recognized as an abundant component of the chromatin [96] and robust evidence underscores the important roles of many ncRNAs in regulating gene expression and shaping genome organization [97]. SnoRNAs have been shown to be particularly enriched in the chromatin of both insect and mammalian cells [3, 33, 56, 98]. Additionally, genome-wide mapping of RNA–DNA interactions using ChAR-seq has identified a significant association between numerous snoRNAs, including *snoRNA:U3:9B*, and protein-coding genes in the chromatin of *D. melanogaster* [3]. These findings suggested that snoRNAs may play a dedicated role in the regulation of gene expression. Here, we demonstrate that the *snoRNA:U3:9B* is upregulated in response to viral infections, plays a crucial role in the activation of immune response genes, and is required for survival during viral infection.

Viruses often hijack the cellular machineries to support their own replication and transcription, and a plethora of mechanisms have been discovered by which ncRNAs become repurposed upon viral infection. A gene-trap insertional mutagenesis study carried out in five mammalian cell lines using a battery of 12 different viruses identified as many as 83 snoRNAs that were required for efficient viral infectivity [99]. Not only snoRNAs, but also miRNAs and lncRNAs have been shown to promote viral replication or suppress the cellular immune response through a variety of molecular mechanisms [42, 100]. Moreover, snoRNA upregulation in response to viral infections is a relatively common phenomenon [101, 102] and thus the fact that *snoRNA:U3:9B* is upregulated in the Sindbis model is not unexpected. The novelty of our current findings lies instead in the identification of *snoRNA:U3:9B* as a critical component of the antiviral response.

We have made use of an already established model for studies of Sindbis replication in *Drosophila* based on the GAL4-induced expression of a viral replicon integrated in the *Drosophila* genome [72]. In this model, the structural capsid proteins are not synthesized, which precludes virion production and the spreading of a systemic infection. However, the expression of the viral nonstructural proteins is efficiently induced and the host gene expression is changed in ways that mimic to a remarkable extent the gene expression changes occurring in a *bona-fide* viral infection. These antiviral responses include not only RNAi, but also activation of specific antimicrobial pathways and induction of immune response genes, as initially shown by Avadhanula *et al.* [72]. Most of our present conclusions are derived from studies in which we used this *SIN* replicon model. However, we have also demonstrated the antiviral role of *snoRNA:U3:9B* in infection experiments using a wild-type, fully-infective strain of Sindbis virus, which reinforces the physiological significance of *snoRNA:U3:9B* in antiviral defence.

Canonical C/D box snoRNAs are associated with a set of snoRNA-binding proteins that typically includes the 2′-O-methyltransferase Fibrillarin. Our FISH experiments showed a broad distribution of *U3 snoRNAs* in salivary gland polytene chromosomes, but failed to reveal a similarly widespread distribution for Fibrillarin. Technical artifacts related to epitope masking are unlikely because the antibody that was used in our experiments was polyclonal. Thus, we favor the interpretation that *snoRNA:U3:9B* is part of a noncanonical snoRNP complex that lacks Fibrillarin. Several mechanisms have been reported by which snoRNAs facilitate gene expression in chromatin through noncanonical pathways [12, 103]. In mammalian cells, a set of snoRNAs interact with the mRNA 3′ processing complex and negatively regulate mRNA 3′ end formation co-transcriptionally by blocking the interaction between the cleavage and polyadenylation specificity factor and the polyadenylation signal in the nascent pre-mRNA [26]. SnoRNAs can also regulate pre-mRNA splicing through RNA–RNA interactions [28]. Furthermore, snoRNAs have been suggested to facilitate the establishment of open chromatin states by binding to histone tails via a tripartite interaction that involves the chromatin decondensation factor Df31 [33].

Our present findings are compatible with a model in which *snoRNA:U3:9B* is targeted to specific genes through RNA:RNA interactions and acts locally to promote an open chromatin conformation that is required for efficient gene activation. Pull-down experiments using antisense oligonucleotide probes revealed a physical association between the *snoRNA:U3:9B* and target immune response genes in the absence of an immune challenge, albeit at relatively low levels. Notably, this association was significantly enhanced in the context of viral replication. We have not formally investigated whether this increased association is directly related to the increased abundance of *snoRNA:U3:9B* in cells that support Sindbis replication or is caused by a specific targeting mechanism. The fact that not all the analyzed target genes show the same increase suggests that the association between the *snoRNA:U3:9B* and a specific subset of target genes relies on dedicated targeting mechanisms that operate in response to infection. Such targeting mechanisms are likely to involve specific interactions and the significant enrichment of *snoRNA:U3:9B* binding sites. The use of a computational tool for prediction of snoRNA targets identified a high number of potential hits for *snoRNA:U3:9B*, and we identified RNA sequence motifs enriched in the immune target transcripts that were predicted to interact with *snoRNA:U3:9B*. Based on these observations, we envision that direct RNA:RNA interactions between *snoRNA:U3:9B* and nascent transcripts could enhance the association of the snoRNA with activated immune response genes early during gene activation.

Viral infections induce the expression of a large number of genes that are regulated through different signaling pathways [44, 48, 49]. The details of such regulation are far from being fully understood and are, to a certain extent, specific for different types of viruses. While specific transcription factors that play pivotal roles in mediating the transcriptional response to immune challenges have been identified and extensively studied [48, 53, 55], the mechanisms by which chromatin regulation shapes the immune transcriptional responses remain less understood. Our current findings reveal that infection with the Sindbis virus induces significant alterations in chromatin accessibility at specific genomic loci. Furthermore, our results indicate that the activation of the antiviral response to Sindbis virus is more complex than previously recognized and involves a novel snoRNA that facilitates the recruitment of Brahma, the ATPase subunit of the SWI/SNF chromatin remodeling complex, to the promoters of specific genes. ChIRP and ChIP-qPCR experiments have shown the co-occupancy of *snoRNA:U3:9B* and Brahma within chromatin. The co-occupancy of *snoRNA:U3:9B* and Brahma was stronger in immune challenged conditions, and *snoRNA:U3:9B* was required for enhanced Brahma occupancy at genes that code for regulatory proteins such as the POU/homeodomain transcription factor Nubbin or the MAP kinase encoded by *rolled (rl)/Erk*. Our study does not distinguish between primary and secondary effects, and the intricate mechanisms by which *snoRNA:U3:9B* contributes to the sequence of events that govern chromatin dynamics during the antiviral response warrants further investigation. Interestingly, the human BRG1 protein -an ortholog of *Drosophila* Brahma- is an RNA-binding protein that interacts with U3 snoRNA [104]. This finding suggests that the involvement of specific snoRNAs in recruiting chromatin remodelers to activate transcription may be conserved across species.

Here we have investigated *snoRNA:U3:9B* and established its role in the activation of immune genes. Notably, two independent methodologies for genome-wide identification of *snoRNA:U3:9B* target genes -namely, an experimental approach utilizing ChAR-seq [3] and *in silico* analyses using target prediction tools [19]- have uncovered associations between *snoRNA:U3:9B* and thousands of genes in the *D. melanogaster* genome. In contrast to the widespread distribution of *snoRNA:U3:9B* across the genome, its deletion only had a modest effect on the transcriptome. A possible explanation for this apparent discrepancy is that *snoRNA:U3:9B* is part of a broad mechanism that helps maintain permissive chromatin states at many loci and is required for efficient gene activation in response to stimuli, but is not required for steady-state transcription. Interestingly, the association of *snoRNA:U3:9B* with protein-coding genes extends beyond genes related to immune responses, highlighting the broad regulatory potential of *snoRNA:U3:9B*. GO enrichment analyses of genes associated with *snoRNA:U3:9B* revealed consistent links to various signaling pathways, and this observation raises the intriguing possibility that snoRNAs may play a general role in enabling inducible chromatin states by facilitating the recruitment of ATP-dependent chromatin remodelers.

## Supplementary Material

gkaf715_Supplemental_Files

## Data Availability

The RNA-seq and ATAC-seq data produced in this study are available from NCBI Gene Expression Omnibus (GEO accession GSE281912).

## Notes

Present address: Instituto de Biotecnología y Biomedicina (BiotecMed)/Departamento de Biología Celular, Universidad de Valencia, Valencia ES-46010, Spain

Present address: Department of Microbiology, Tumor and Cell Biology, Karolinska Institute, Solna SE-171 65, Sweden
