# Supplementary material for: A U3 snoRNA is required for the regulation of chromatin dynamics and antiviral response in Drosophila melanogaster
Source: Nucleic Acids Res. 2025 Jul 30;53(14):gkaf715. doi: 10.1093/nar/gkaf715 (PMC12309367; doi:10.1093/nar/gkaf715)
Supplement: gkaf715_Supplemental_Files [file gkaf715_supplemental_files.zip › Jain et al_Supplementary Data.pdf]

SUPPLEMENTARY DATA

A U3 snoRNA is required for the regulation of chromatin dynamics and antiviral response in Drosophila melanogaster

Jain et al.

Supplementary figures

Supplementary FigureS1	SnoRNA:U3 sequences in *Drosophila melanogaster*
Supplementary FigureS2	Characterization of ca-snoRNAs in *Drosophila melanogaster*
Supplementary FigureS3	Distribution of *snoRNA:185* binding sites in gene features
Supplementary FigureS4	Analysis of snoRNA expression in virus infected larvae
Supplementary Figure S5	*SIN* replicon expression induces the expression of immune response genes in the cardia of third instar larvae
Supplementary FigureS6	*SIN* replicon expression in *snoRNA:U3:9B* knockouts
Supplementary FigureS7	Transcriptome analysis of third instar larval brain in wild-type and *snoRNA:U3:9B* knockout larvae
Supplementary FigureS8	Chromatin accessibility analysis of third instar larval brain in wild-type and *snoRNA:U3:9B* knockout larvae
Supplementary FigureS9	Chromatin accessibility analysis of third instar larval brain expressing *SIN* replicon in *snoRNA:U3:9B* knockout larvae compared to wild-type
Supplementary FigureS10	The immune response of S2 cells to Sindbis expression is inhibited by *snoRNA:U3:9B* depletion
Supplementary FigureS11	*SnoRNA:185* physically interacts with target immune response genes
Supplementary FigureS12	Genome wide prediction of snoRNA:target RNA interactions using snoGloBe
Supplementary FigureS13	*SnoRNA:U3:9B* is required for the recruitment of the ATP-dependent chromatin remodeller Brahma to target immune genes

Supplementary references

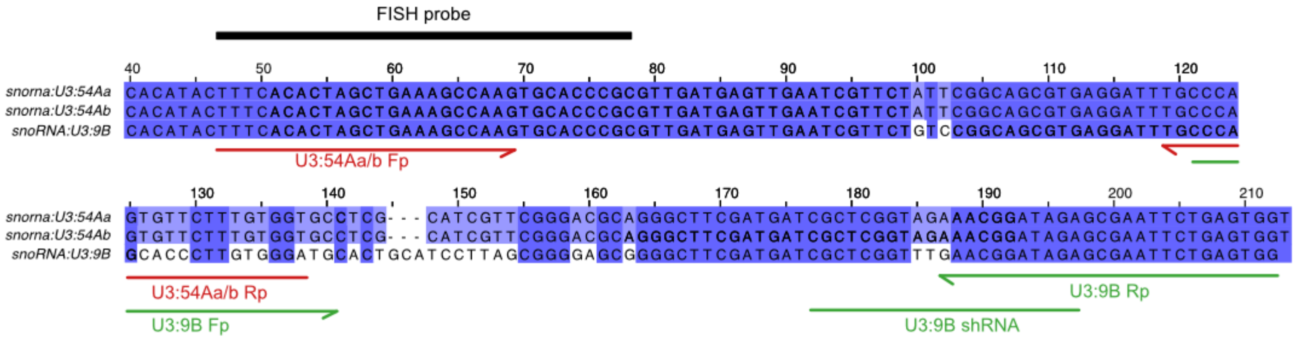

Figure S1. *SnoRNA:U3* sequences in *Drosophila melanogaster*
Three U3 snoRNA paralogues exist in *D. melanogaster*. The three U3 snoRNAs have been recently renamed in FlyBase and are now referred to as *snoRNA:CD11a,b,c*. *SnoRNA:U3:9B* is *snoRNA:CD11c*. The figure shows a multiple sequence alignment of the three U3 snoRNA paralogues and indicates the positions of the probes and primers used in this study. Primer and probe sequences are also provided in Supplementary Table S1.

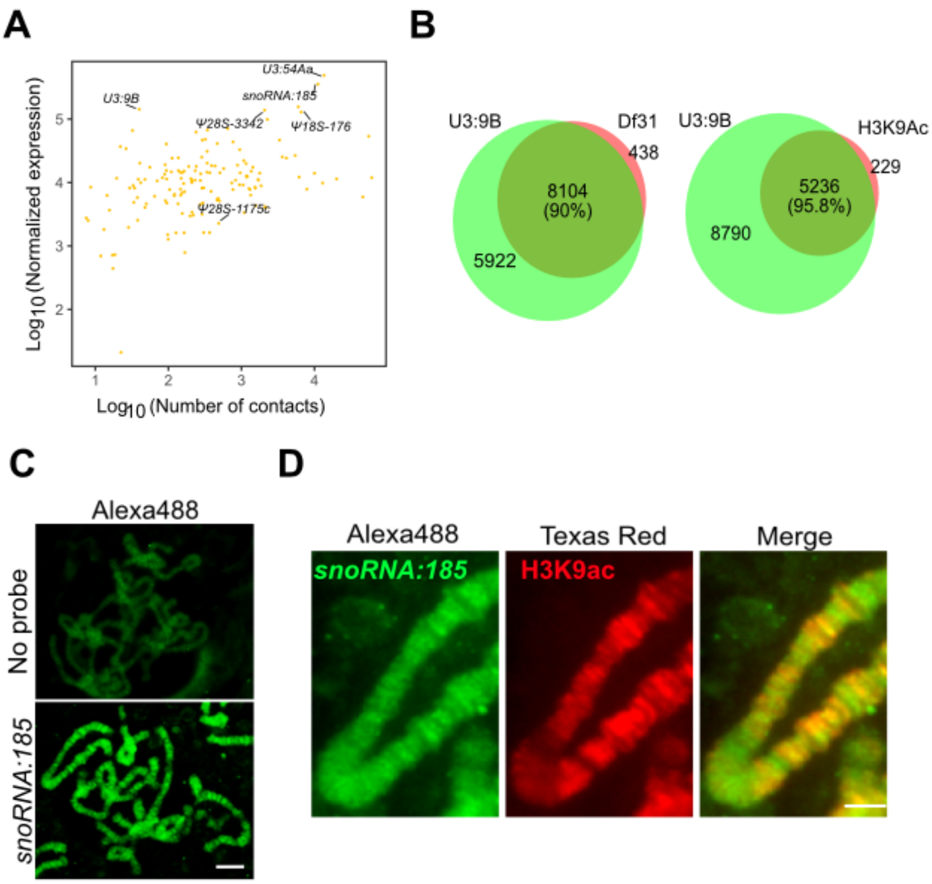

Figure S2. Characterization of ca-snoRNAs in *Drosophila melanogaster*

A. Scatter plot comparing the number of contacts identified by ChAR-seq (x axis) with the normalized expression (RNA-seq, y axis). Data from Bell et al. (2018) (1). Some chromatin enriched ca-snoRNAs are highlighted.

B. Venn diagrams showing the overlap between ChAR-seq contacts for *snoRNA:U3:9B* from Bell et al. (1) and either the Df31 bound genes identified by DamID in Kc167 cells by Filion et al. (2) (left hand side) or the H3K9ac peaks identified by ChIP-seq (1) (right hand side).

C. RNA-FISH showing the distribution of snoRNA:185 (green, lower panel) in polytene chromosomes of salivary glands of third instar larvae. The scale bar represents approximately 20 μm.

D. RNA-FISH showing the co-localization of H3K9ac (Texas red) with DIG labeled *snoRNA:185* (Alexa488) in polytene chromosomes of salivary glands of third instar larvae. The scale bar represents approximately 10 μm.

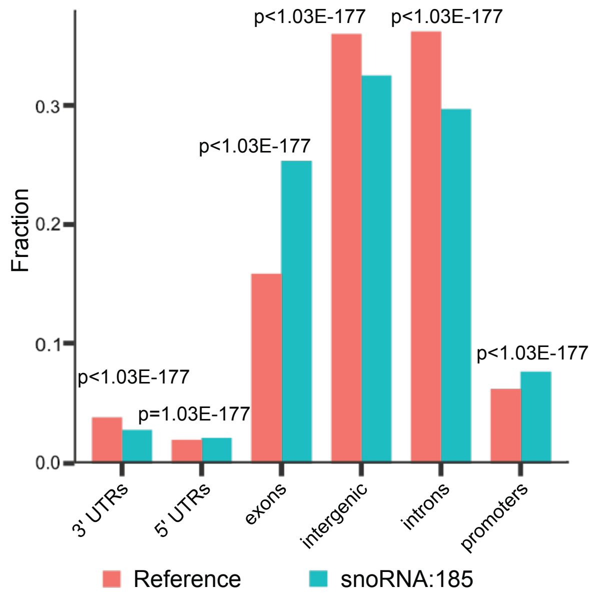

Figure S3. Distribution of *snoRNA:185* binding sites in gene features

Meta-analysis of ChAR-seq data (1). The bar plot shows the distribution of snoRNA:185 contact sites (blue) in specific gene feature as compared to the reference genome distribution (red). 2-sample proportional z-test with Benjamini-Hochberg correction was used to compare the distributions.

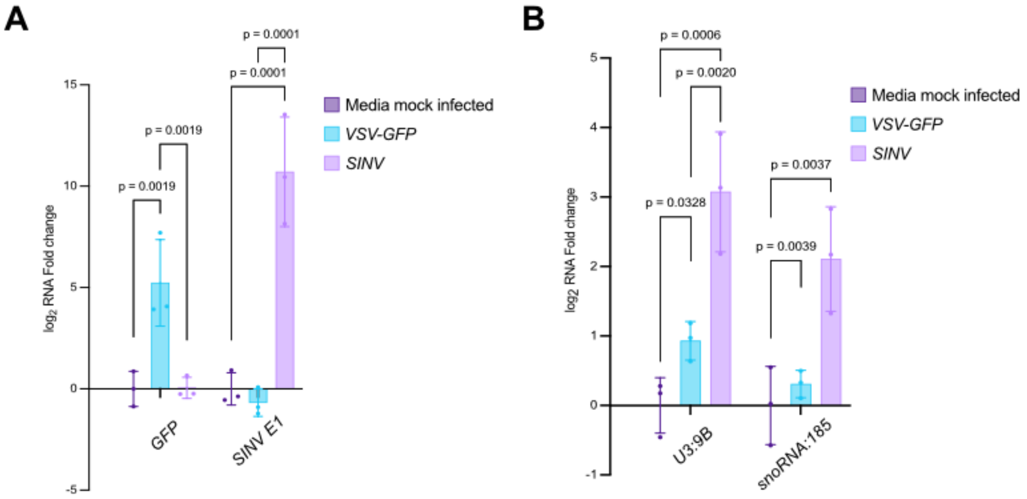

Figure S4. Analysis of snoRNA expression in virus infected larvae

Sindbis virus (SINV) or vesicular stomatitis virus (VSV-GFP) preparations were injected into third instar larvae. RNA was extracted from larval brains dissected 24 hr after infection.

A. The specificity of the infections was analyzed by RT-qPCR to quantify the expression of viral sequences. PCR primers specific for GFP or SINV-E1 were used to detect VSV-GFP and SINV, respectively.

B. SnoRNAs expression was analyzed by RT-qPCR.

In all cases, PCR results were normalized to Act5C. Mock infected animals were analyzed in parallel. The RNA levels are expressed as log2 fold change compared to mock infected controls. Multiple comparisons were carried out using ordinary one-way ANOVA combined with a two-stage step-up procedure of Benjamini, Krieger and Yekutieli. Adjusted p-values are shown in the figure. N=3.

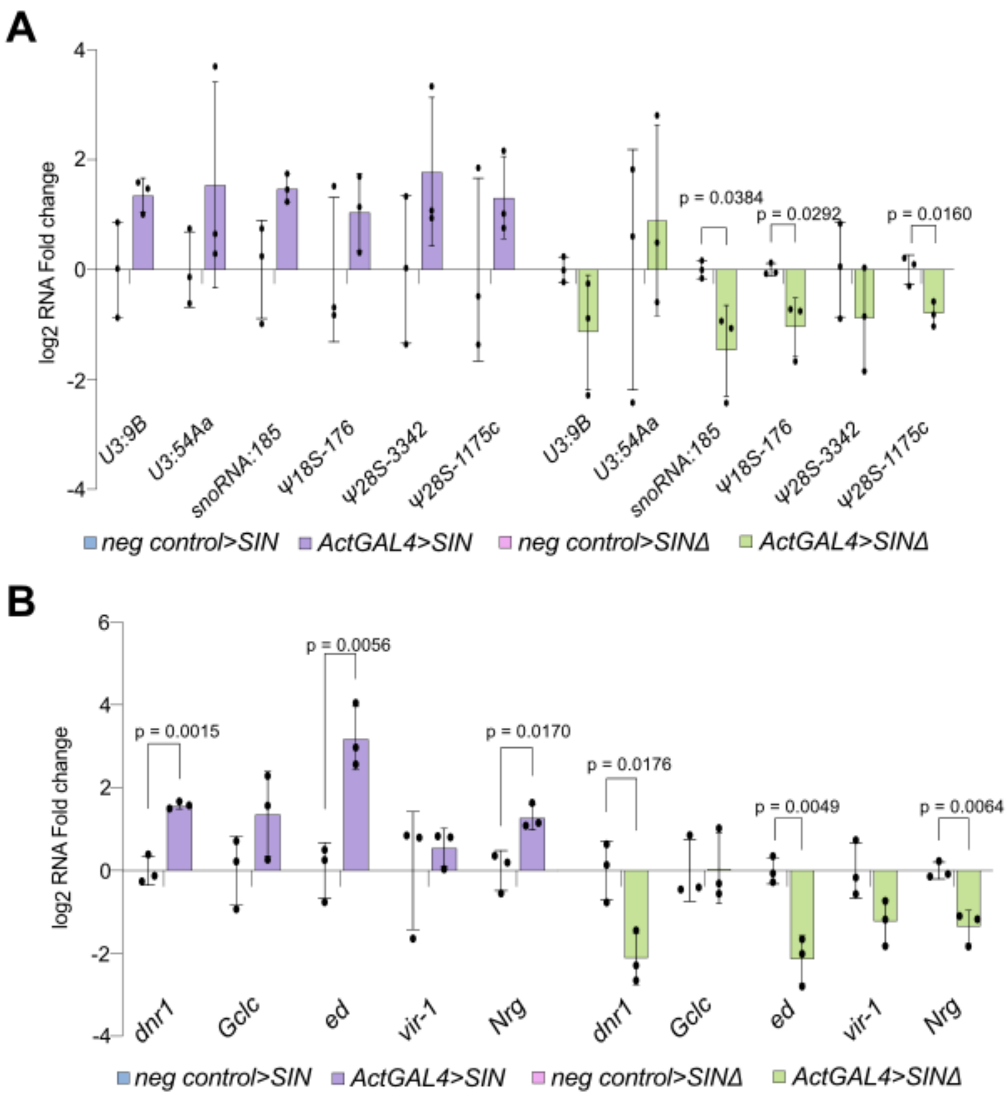

Figure S5. *SIN* replicon expression induces the expression of immune response genes in the cardia of third instar larvae

- **A.** Bar plot showing the induction of ca-snoRNAs in cardia of *SIN* third instar larvae with (purple) and without (blue) *ActGAL4* driver (N=5). The graph also shows the lack of induction of ca-snoRNAs in cardia of *SIN∆* larvae with and without *ActGAL4* driver (green and pink, respectively). The expression was measured by RT-qPCR and normalized to *Act5C*. The RNA levels are expressed as $\log_2$ fold change compared to controls. A two tailed Student's unpaired t-test was used to compare the data sets. N=3
- **B.** Bar plot showing the induction of target immune response genes in the same four conditions described in (**a**). The expression was measured by RT-qPCR and normalized to *Act5C*. A two tailed Student's unpaired t-test was used to compare the data sets.

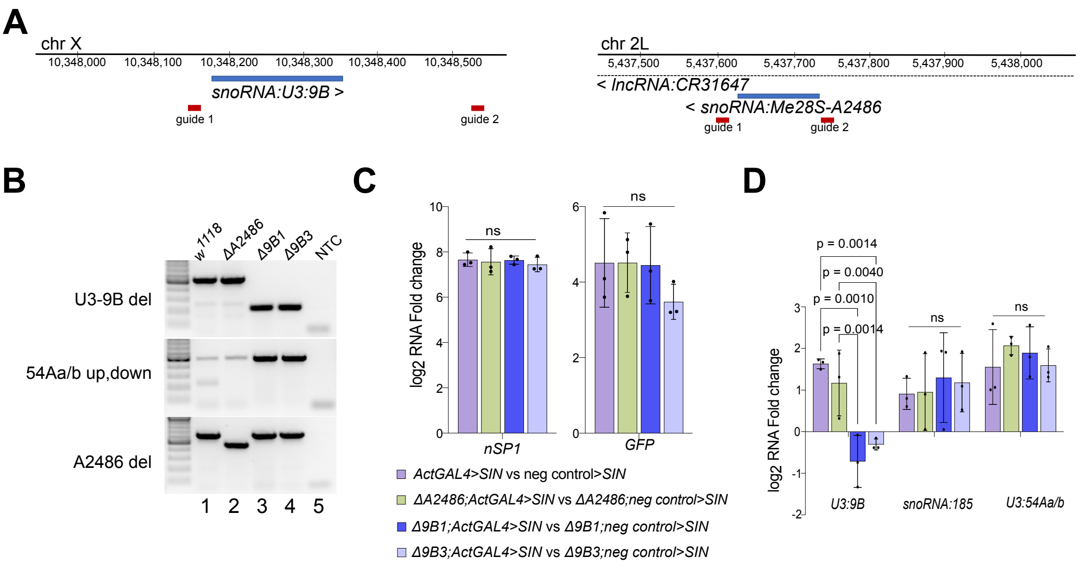

Figure S6. *SIN* replicon expression in *snoRNA:U3:9B* knockouts

A. Schematic showing the position of two gRNAs (red boxes) targeting *snoRNA:U3:9B* (left) and *snoRNA:Me28S-A2486* (right). The *snoRNA:U3:9B* gene is an independent gene while *snoRNA:Me28S-A2486* is intronic.

B. Agarose gel electrophoresis showing PCR amplicons obtained from single fly genomic DNA. The lower molecular mass bands for *snoRNA:U3:9B* in lanes 3 and 4 (top panel) compared to w^{1118} and for *snoRNA:Me28S-A2486* in lane 2 (bottom panel) indicate successful deletion of the corresponding snoRNA. The specificity of the deletions is shown by PCR using primers targeting another U3 snoRNA paralogue, *snoRNA:U3:54Aa/b* (middle panel).

C. Bar plot showing the expression of the *SIN* replicon (*nSP1* expression, left and *GFP*, right) in brains of third instar wild-type larvae (*ActGAL4>SIN* vs *neg control>SIN*), control snoRNA knock-out (*ΔA2486; ActGAL4>SIN* vs *ΔA2486; neg control>SIN*) or *snoRNA:U3:9B* knock-out (*Δ9B1; ActGAL4>SIN* vs *Δ9B1; neg control>SIN* and *Δ9B3; ActGAL4>SIN* vs *Δ9B3; neg control>SIN*). The expression of *nSP1* and *GFP* was measured by RT-qPCR, normalized to *Act5C* and expressed as $\log_2$ fold change relative to the expression in brain dissected from larvae without GAL4. Multiple comparisons were carried out using ordinary one-way ANOVA combined with a two-stage step-up procedure of Benjamini, Krieger and Yekutieli. Adjusted p-values are shown. N=3

D. Bar plot showing the expression of different snoRNAs in brains of third instar wild-type larvae (*ActGAL4>SIN* vs *neg control>SIN*), control snoRNA knock-out (*ΔA2486; ActGAL4>SIN* vs *ΔA2486; neg control>SIN*) or *snoRNA:U3:9B* knock-out (*Δ9B1; ActGAL4>SIN* vs *Δ9B1; neg control>SIN* and *Δ9B3; ActGAL4>SIN* vs *Δ9B3; neg control>SIN*). The expression was measured by RT-qPCR, normalized to *Act5C* and expressed as $\log_2$ fold change relative to signal in control brains without GAL4. Multiple comparisons were carried out using ordinary one-way ANOVA combined with a two-stage step-up procedure of Benjamini, Krieger and Yekutieli. Adjusted p-values are shown. N=3. Legends as in C.

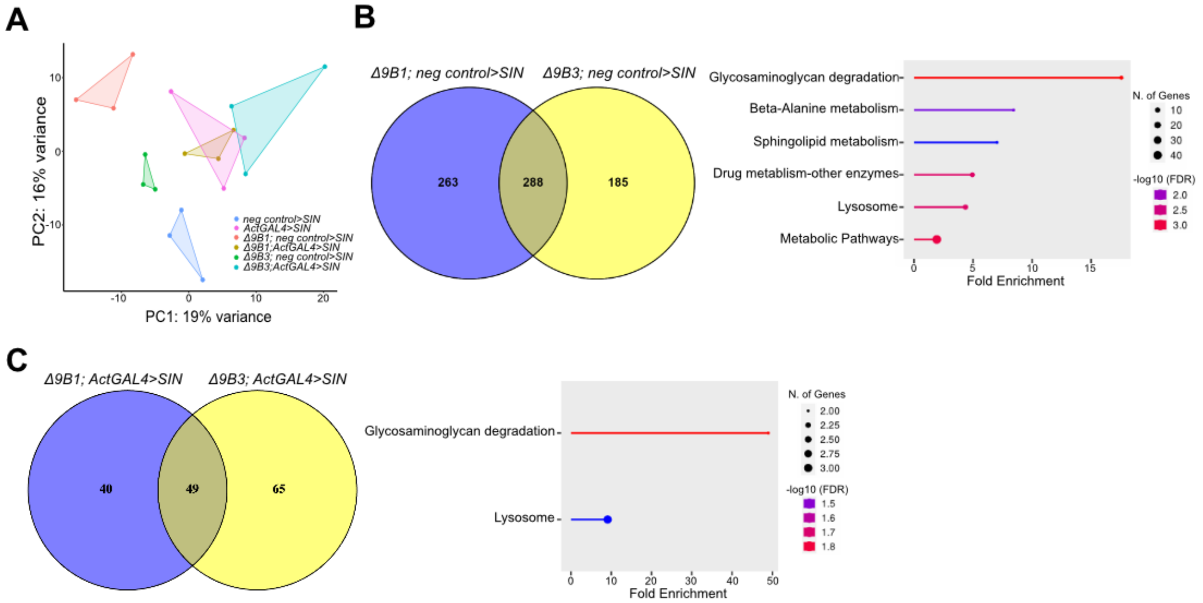

Figure S7. Transcriptome analysis of third instar larval brain in wild-type and *snoRNA:U3:9B* knockout larvae

A. Principal component analysis of gene expression profiled by RNAseq in dissected brains of third instar larvae of wild-type (*SIN*) and *snoRNA:U3:9B* knockouts (*Δ9B1;SIN* and *Δ9B3;SIN*) with and without *ActGAL4* driver. The three replicates of each condition are represented in the plot. The replicates from the same condition cluster together and the transcriptome varies in samples with and without *SIN* replicon expression.

B. Intersection (left) of differentially expressed transcripts ($p_{adj} < 0.05$) detected by differential expression analysis in *snoRNA:U3:9B* knockouts (*Δ9B1; neg control>SIN* and *Δ9B3; neg control>SIN*) compared to wild-type (*neg control>SIN*), without *ActGAL4* driver. KEGG GO classification (right) of 288 differentially expressed transcripts common to both deletion lines. The histone transcripts were removed from the analysis.

C. Intersection (left) of significantly differentially expressed transcripts ($p_{adj} < 0.05$) detected by differential expression analysis in *SIN* replicon expressing larvae with *snoRNA:U3:9B* knockouts (*Δ9B1; ActGAL4>SIN* and *Δ9B3; ActGAL4>SIN*) as compared to wild-type condition (*ActGAL4>SIN*). KEGG GO classification (right) of 49 differentially expressed transcripts common to both deletion lines. The histone transcripts were removed from the analysis.

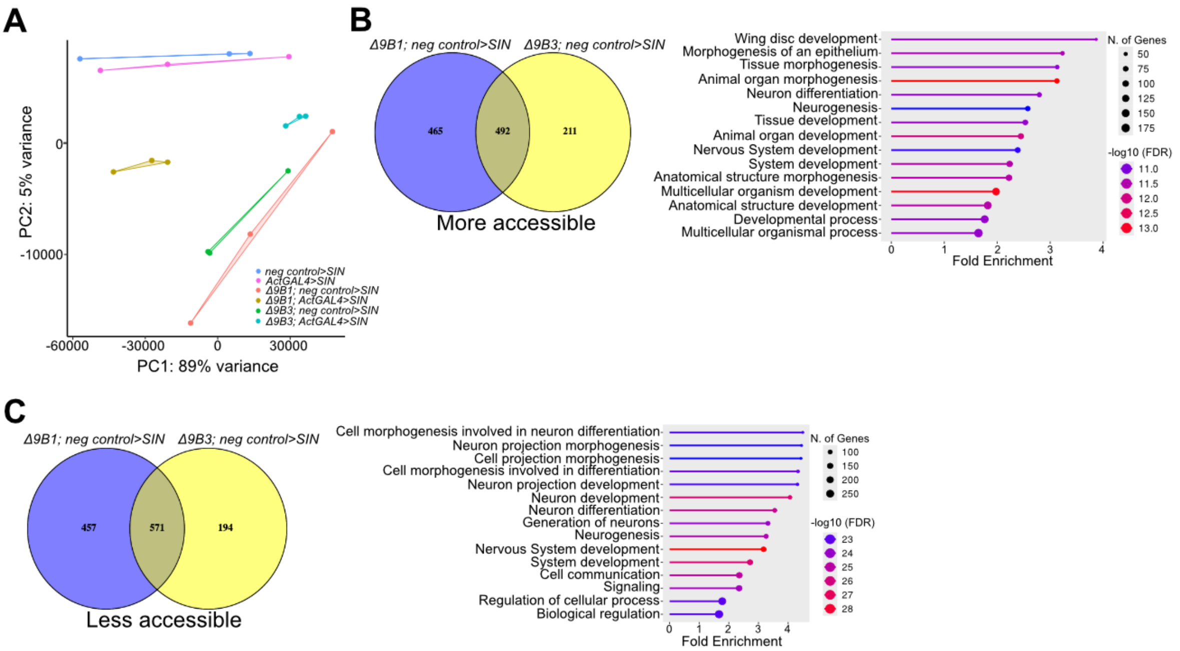

Figure S8. Chromatin accessibility analysis of third instar larval brain in wild-type and *snoRNA:U3:9B* knockout larvae

A. Principal component analysis of ATAC-seq data from wild-type (*SIN*) and *snoRNA:U3:9B* knockout (*Δ9B1;SIN* and *Δ9B3;SIN*) third instar larval brain, with and without *ActGAL4* driver. The three replicates of each condition are represented in the plot. Note that the replicates from the same condition cluster together and that the transcriptome varies between samples wild-type and *snoRNA:U3:9B* knockout.

B. Intersection (left) of genes that contain at least one significantly more accessible region detected by edgeR analysis ($p_{adj} < 0.05$) in *snoRNA:U3:9B* knockouts (*Δ9B1; neg control*>*SIN* and *Δ9B3; neg control*>*SIN*) compared to wild-type (*neg control*>*SIN*). GO enrichment test for biological process (right) of the 492 genes that contain at least one significantly more accessible region in both deletion lines.

C. Intersection (left) of genes that contain at least one significantly less accessible region detected by edgeR analysis ($p_{adj} < 0.05$) in *snoRNA:U3:9B* knockouts (*Δ9B1; neg control*>*SIN* and *Δ9B3; neg control*>*SIN*) compared to wild-type (*neg control*>*SIN*). GO enrichment test for biological process (right) of the 571 differentially accessible regions in genes common to both deletion lines.

Some genes included regions with increased accessibility and regions with reduced accessibility. These genes are counted in both **B.** and **C.** The total number of affected genes is 978.

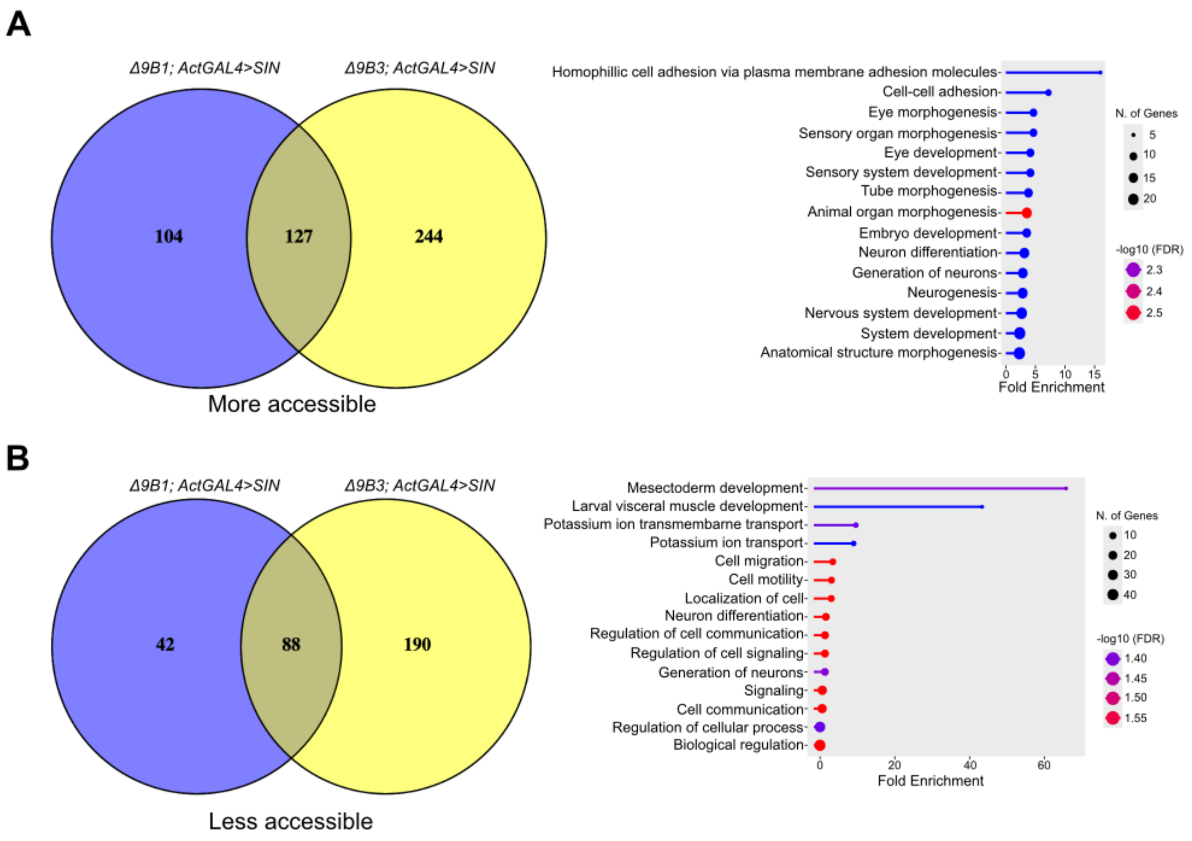

Figure S9. Chromatin accessibility analysis of third instar larval brain expressing *SIN* replicon in *snoRNA:U3:9B* knockout larvae compared to wild-type

A. Intersection (left) of genes that contain at least one more accessible region detected by edgeR analysis ($p_{adj} < 0.05$) in *SIN* replicon expressing larvae with *snoRNA:U3:9B* knockouts (*Δ9B1; ActGAL4>SIN* and *Δ9B3; ActGAL4>SIN*) compared to wild-type (*ActGAL4>SIN*). GO enrichment test for biological process (right) of the 127 genes with at least one more accessible region common to both deletion lines.

B. Intersection (left) of genes associated with significantly less accessible regions detected by edgeR analysis ($p_{adj} < 0.05$) in *SIN* replicon expressing larvae with *snoRNA:U3:9B* knockouts (*Δ9B1; ActGAL4>SIN* and *Δ9B3; ActGAL4>SIN*) compared to wild-type (*ActGAL4>SIN*). GO enrichment test for biological process (right) of the 88 genes with less accessible regions common to both deletion lines.

Some genes included regions with increased accessibility and regions with reduced accessibility. These genes are counted in both **A.** and **B.** The total number of affected genes is 201.

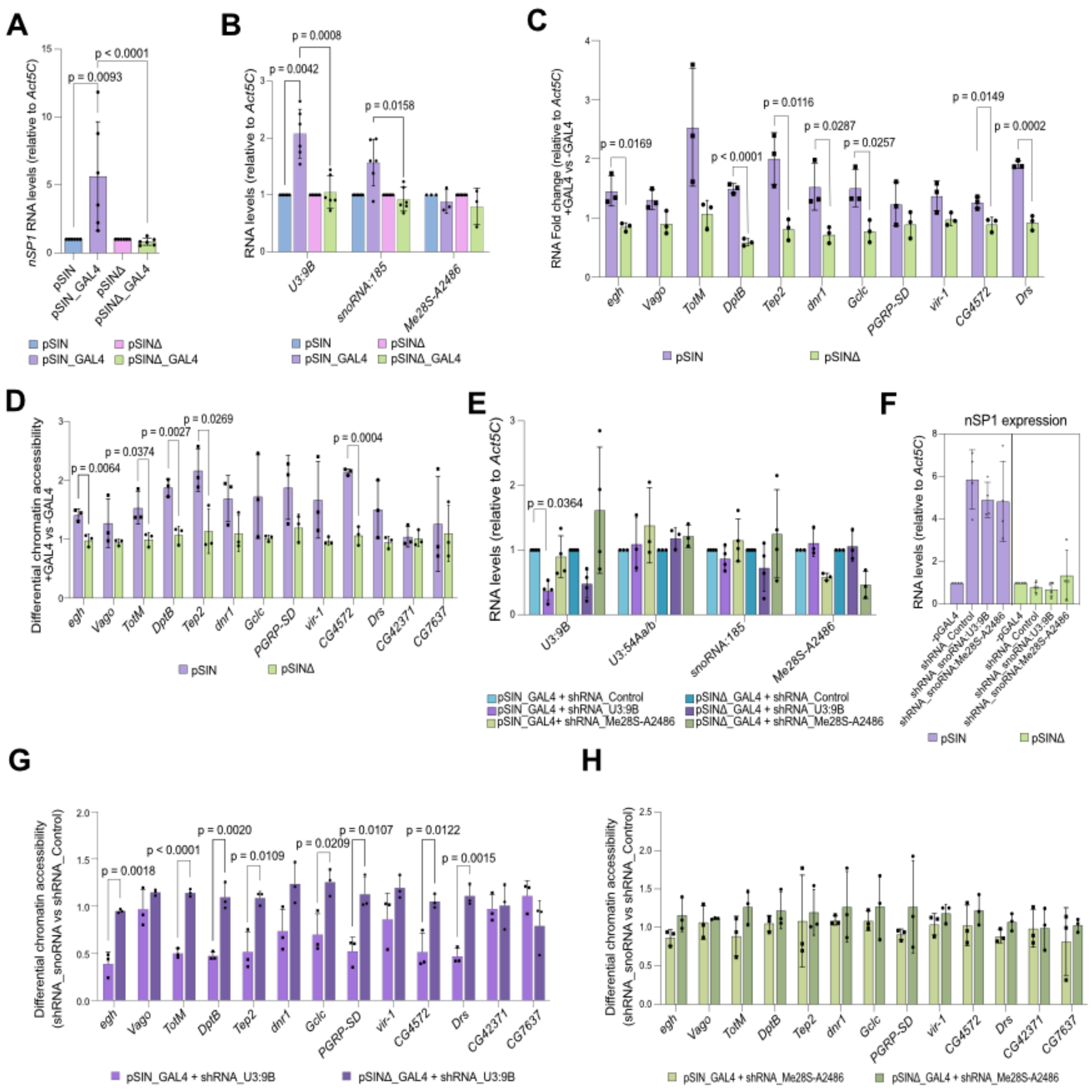

Figure S10. The immune response of S2 cells to Sindbis expression is inhibited by *snoRNA:U3:9B* depletion

A. Bar plot showing *nSP1* relative mRNA levels in S2 cells stably transfected with viral replicon pSIN_GAL4 (purple bar) and pSIN cells not expressing the replicon (blue bar). pSINΔ and pSINΔ_GAL4 cells (pink and green bars, respectively) were also analyzed in parallel. The expression was measured by RT-qPCR and normalized to *Act5C*. The analysis was carried out 48 hr after transfection of the pAc-GAL4 construct to drive the expression of the viral replicon. Statistical significance was tested using a non-parametric Kruskal-Wallis rank sum test combined with a two-stage step-up procedure of Benjamini, Krieger and Yekutieli. Adjusted p-values are shown. N=6.

B. Bar plot showing relative snoRNA levels in S2 cells expressing the *SIN* replicon (pSIN_GAL4, purple bar) as compared to cells not expressing the replicon (pSIN, blue bar). The comparison is also made to S2 cells carrying the pSINΔ replicon, with and without GAL4 induction (green and

pink bars, respectively). The expression was measured by RT-qPCR and normalized to *Act5C*. The S2 cells stably transfected with viral replicon were transiently transfected with the pAc-GAL4 construct to drive the expression of viral replicon and harvested for RT-qPCR analysis 48 hr after transfection. Statistical significance was tested using a non-parametric Kruskal-Wallis rank sum test combined with a two-stage step-up procedure of Benjamini, Krieger and Yekutieli. Adjusted p-values are shown. N=6.

C. Bar plot showing changed expression of *snoRNA:U3:9B* target immune genes in response to *SIN* replicon expression. The expression of selected target genes was measured by RT-qPCR and normalized to *Act5C* in the same four conditions described in (**b**): pSIN cells with or without pAc-GAL4 and pSINΔ cells with or without pAc-GAL4. The plot shows the fold changes of normalized RNA levels in pSIN_GAL4 (purple bars) and pSINΔ_GAL4 (green bars) cells compared to the respective uninduced controls (without pAc-GAL4). Two tailed Student's unpaired t-test was used to compare the data sets. N=3

D. Bar plot showing the differential chromatin accessibility of *snoRNA:U3:9B* bound genes in pSIN cells (purple bars) and pSINΔ cells (green bars) as measured by ATAC-qPCR and normalized to *Ctp*. The differential accessibility is expressed relative to that of control cells without induction of viral replicon (without pAc-GAL4). Two tailed Student's unpaired t-test was used to compare the data sets. N=3

E. Bar plot showing the knockdown of different snoRNAs in S2 cells. The relative RNA levels for each snoRNA in the shRNA transfected cells were measured by RT-qPCR 48 h after transfection, normalized to *Act5C* and expressed relative to the expression in cells transfected with shRNA_control. Statistical significance was tested using a non-parametric Kruskal-Wallis rank sum test combined with a two-stage step-up procedure of Benjamini, Krieger and Yekutieli. Adjusted p-values are shown. N=4

F. Bar plot showing the relative *nSP1* expression in S2 cells expressing pSIN (purple bars) and in pSINΔ cells (green bars) transfected with different shRNAs. The cells were transfected with different shRNA constructs, as indicated, and *nSP1* expression is expressed relative to -pGAL4 cells (first bar of each replicon). There were no significant differences in *nSP1* expression among cells transfected with different shRNAs. *nSP1* expression was significantly higher (p_{adj} = 0.0261) in pSIN_GAL4 cells (irrespective of shRNA construct) than in pSINΔ cells. In all cases, RNA levels were measured by RT-qPCR 48 hr after transfection of the corresponding shRNA construct and pAc-GAL4, and normalized to *Act5C*. Nested t-test was used for statistical testing. N=4

G. Depletion of *snoRNA:U3:9B* in S2 cells expressing pSIN abolishes chromatin accessibility changes at *snoRNA:U3:9B* target genes. The bar plot shows the effect of *snoRNA:U3:9B* depletion on chromatin accessibility at *snoRNA:U3:9B* target gene loci in S2 cells expressing pSIN_GAL4 (as compared to cells expressing pSINΔ_GAL4). Chromatin accessibility was measured by ATAC-qPCR, normalized to *Ctp* and expressed relative to control cells expressing control shRNA along with different viral replicons. Two tailed Student's unpaired t-test was used to compare the data sets. N=3

H. Depletion of *snoRNA:Me28S-A2486* in S2 cells expressing pSIN does not affect chromatin accessibility at *snoRNA:U3:9B* target genes. Bar plot showing the differential chromatin accessibility of *snoRNA:U3:9B* target genes in *snoRNA:Me28S-A2486* depleted cells expressing pSIN_GAL4 (as compared to cells expressing pSINΔ_GAL4). Chromatin accessibility analysis and statistical testing as in (**g**). N=3

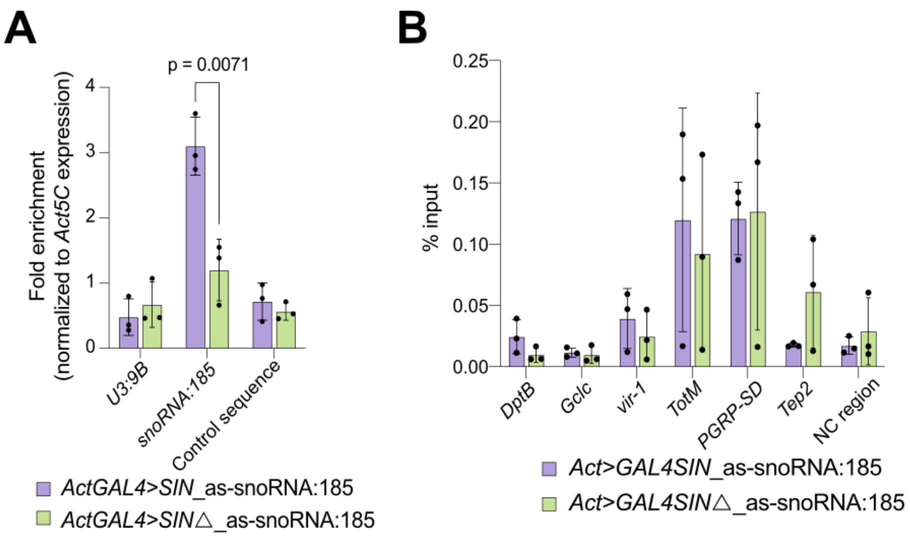

Figure S11: *SnoRNA:185* physically interacts with target immune response genes

A. Bar plot showing ChIRP specificity. *SnoRNA:U3:9B*, *snoRNA:185* and a control unrelated sequence were quantified by RT-qPCR in RNA samples isolated from larval brain tissue from *ActGAL4>SIN* (purple) and *ActGAL4>SINΔ* (green) by ChIRP using biotinylated DNA probes complementary to *snoRNA:185* (as-snoRNA:185). A two tailed Student's unpaired t-test was used to compare the data sets. N=3

B. Bar plot showing the association of *snoRNA:185* with specific target genes. DNA isolated by ChIRP was analyzed by qPCR using primers for the indicated genes. ChIRP was performed using chromatin from dissected larval brain samples from *ActGAL4>SIN* (purple) and *ActGAL4>SINΔ* (green), as indicated. A two tailed Student's unpaired t-test was used to compare the data sets. N=3

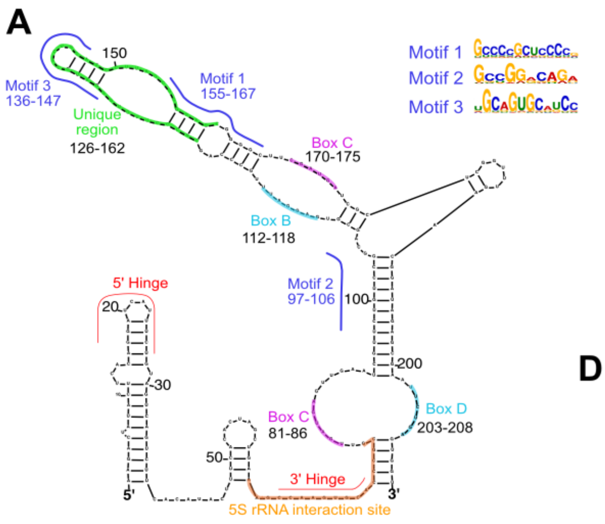

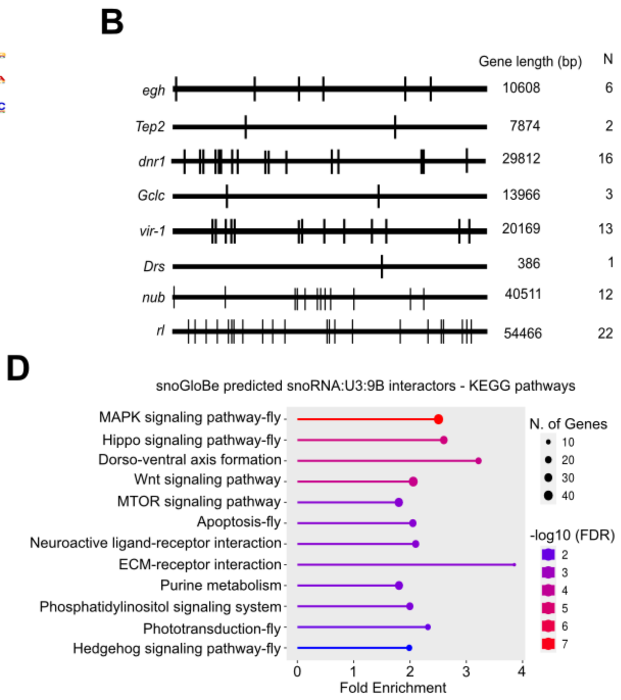

C

SnoRNA name	No. of genes with >5 ChAR-seq binding sites	No. of genes with >5 snoGloBe predicted interactions
SnoRNA:U3:9B	4122	2857
SnoRNA:185	7280	212
SnoRNA:Me28S-A2486	-	833

Figure S12. Genome wide prediction of snoRNA:target RNA interactions using snoGloBe

A. Schematic representation of *snoRNA:U3:9B*. The structure is as predicted in R2DT (3). Predicted boxes C and D/B are highlighted in pink and light blue, respectively. The canonical rRNA interaction sites predicted for U3 snoRNAs are highlighted with red lines representing the 5' and 3' hinge. SnoGloBe (4) predicted an interaction with 5S rRNA (highlighted in orange). The *snoRNA:U3:9B* unique sequence is highlighted in green. The three motifs shown in the figure (highlighted with blue lines) are the most significant motifs identified by STREME in the target sites predicted by snoGlobe for *snoRNA:U3:9B* in the *D. melanogaster* transcriptome (Supplementary Table S6).

B. Schematic of the relative position of *snoRNA:U3:9B* interaction sites predicted by snoGloBe in a subset of immune response genes analysed in the present study. N = number of interaction sites identified with snoGloBe threshold score > 0.98.

C. Table showing the number of genes with >5 contact sites identified by ChAR-seq and number of genes with >5 interactions predicted by snoGloBe for different snoRNAs, as indicated.

D. Gene ontology enrichment analysis of KEGG pathways for transcripts predicted by snoGloBe to have >5 interaction sites for *snoRNA:U3:9B*.

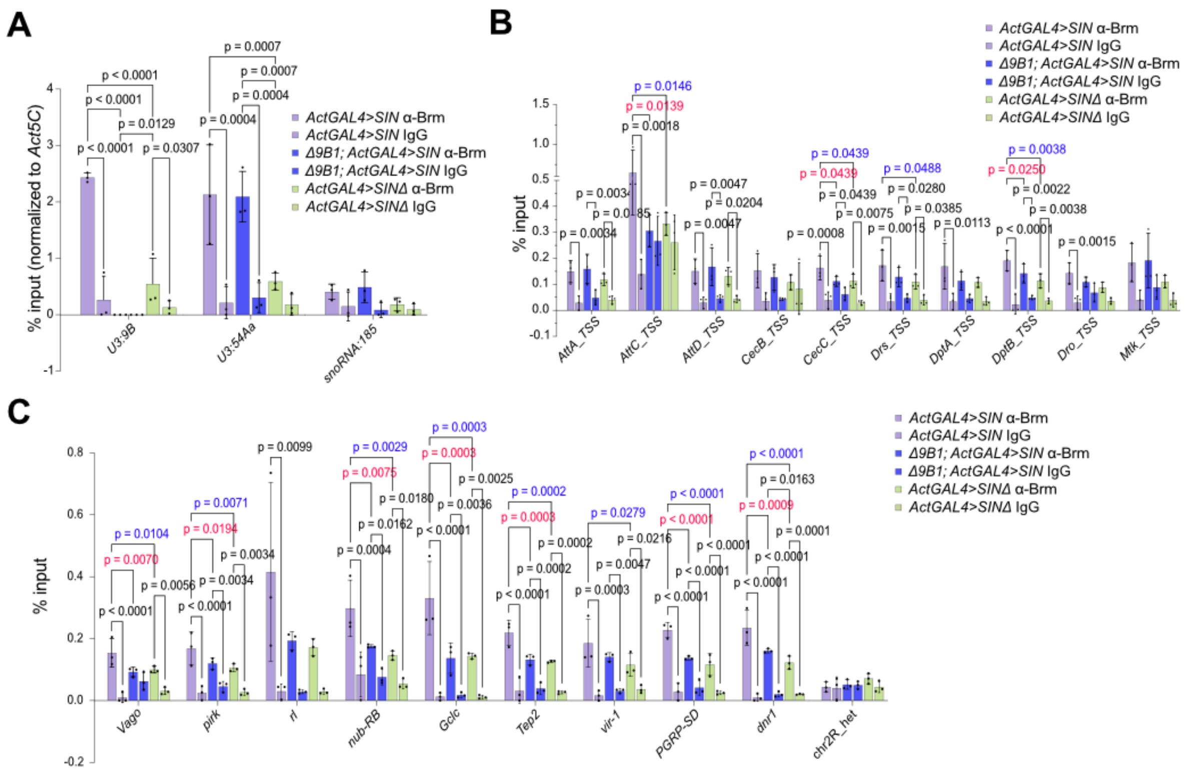

Figure S13. *SnoRNA:U3:9B* is required for the recruitment of the ATP-dependent chromatin remodeller Brahma to target immune genes

A. RT-qPCR analysis of RNA pulled down using ChIP with an antibody against Brg1 that cross-reacts with the *Drosophila* Brahma protein to analyse the co-occupancy of Brahma with ca-snoRNAs in third instar larval brains from wild-type (*ActGAL4>SIN*), *snoRNA:U3:9B* knockout (*Δ9B1; ActGAL4>SIN*) and control *ActGAL4>SINΔ* lines. An IgG antibody was used in parallel as a negative control. The RT-qPCR signals were normalized to the levels of *Act5C.* Multiple comparisons were carried out using ordinary one-way ANOVA combined with a two-stage step-up procedure of Benjamini, Krieger and Yekutieli. Adjusted p-values are shown. N=3

B. ChIP-qPCR using the same antibody as in (**a**) to analyze Brahma occupancy at the TSS of genes coding for AMPs. ChIP was performed in third instar larval brains from wild-type (*ActGAL4>SIN*), *snoRNA:U3:9B* knockout (*Δ9B1; ActGAL4>SIN*) and control *ActGAL4>SINΔ* strains. An IgG antibody was used in parallel as a negative control. Multiple comparisons were carried out using ordinary one-way ANOVA combined with a two-stage step-up procedure of Benjamini, Krieger and Yekutieli. Adjusted p-values are shown. N=3

C. ChIP-qPCR as in (**b**) and analysis of Brahma occupancy at *snoRNA:U3:9B* target genes. The figure presents the same data as in Figure 6e showing the results obtained with the negative control antibody IgG.

Supplementary references

1. Bell,J.C., Jukam,D., Teran,N.A., Risca,V.I., Smith,O.K., Johnson,W.L., Skotheim,J.M., Greenleaf,W.J. and Straight,A.F. (2018) Chromatin-associated RNA sequencing (ChAR-seq) maps genome-wide RNA-to-DNA contacts. *eLife*, **7**, e27024.

2. Filion,G.J., Van Bemmel,J.G., Braunschweig,U., Talhout,W., Kind,J., Ward,L.D., Brugman,W., De Castro,I.J., Kerkhoven,R.M., Bussemaker,H.J., *et al.* (2010) Systematic Protein Location Mapping Reveals Five Principal Chromatin Types in Drosophila Cells. *Cell*, **143**, 212–224.

3. Sweeney,B.A., Hoksza,D., Nawrocki,E.P., Ribas,C.E., Madeira,F., Cannone,J.J., Gutell,R., Maddala,A., Meade,C.D., Williams,L.D., *et al.* (2021) R2DT is a framework for predicting and visualising RNA secondary structure using templates. *Nat Commun*, **12**, 3494.

4. Deschamps-Francoeur,G., Couture,S., Abou-Elela,S. and Scott,M.S. (2022) The snoGloBe interaction predictor reveals a broad spectrum of C/D snoRNA RNA targets. *Nucleic Acids Research*, **50**, 6067–6083.
